# Analysis Transcriptome and Phytohormone Changes Associated with the Allelopathic Effects of Ginseng Hairy Roots Induced by Different-Polarity Ginsenoside Components

**DOI:** 10.3390/molecules29081877

**Published:** 2024-04-19

**Authors:** Tingting Zhou, Qiong Li, Xin Huang, Changbao Chen

**Affiliations:** 1 Jilin Ginseng Academy, Changchun University of Chinese Medicine, Changchun 130117, China; 2 School of Medical Technology, Beihua University, Jilin 132013, China

**Keywords:** metabolome and transcriptome, ginsenoside polarity, allelopathic autotoxicity, molecular mechanism

## Abstract

The allelopathic autotoxicity of ginsenosides is an important cause of continuous cropping obstacles in ginseng planting. There is no report on the potential molecular mechanism of the correlation between polarity of ginsenoside components and their allelopathic autotoxicity. This study applied a combination of metabolomics and transcriptomics analysis techniques, combined with apparent morphology, physiological indexes, and cell vitality detection of the ginseng hairy roots, through which the molecular mechanism of correlation between polarity and allelopathic autotoxicity of ginsenosides were comprehensively studied. The hairy roots of ginseng presented more severe cell apoptosis under the stress of low-polarity ginsenoside components (ZG70). ZG70 exerted allelopathic autotoxicity by regulating the key enzyme genes of cis-zeatin (cZ) synthesis pathway, indole-3-acetic acid (IAA) synthesis pathway, and jasmonates (JAs) signaling transduction pathway. The common pathway for high-polarity ginsenoside components (ZG50) and ZG70 to induce the development of allelopathic autotoxicity was through the expression of key enzymes in the gibberellin (GA) signal transduction pathway, thereby inhibiting the growth of ginseng hairy roots. cZ, indole-3-acetamid (IAM), gibberellin A1 (GA1), and jasmonoyl-L-isoleucine (JA-ILE) were the key response factors in this process. It could be concluded that the polarity of ginsenoside components were negatively correlated with their allelopathic autotoxicity.

## 1. Introduction

*Panax ginseng* C.A. Meyer (ginseng) is a perennial herbaceous medicinal herb with reliable therapeutic effects and has excellent performance in the field of traditional Chinese medicine [1,2]. However, continuous cropping obstacles is always a key factor that affects the high-quality development of ginseng in the industry. Continuous cropping obstacles is a complex phenomenon involving multiple factors such as biotic stress (biological barriers), abiotic stress (autotoxicity, deterioration of soil physical and chemical properties, and imbalance of soil fertility), and their corresponding comprehensive stresses [3]. In the case where the land is continuously used for ginseng replanting, three major diseases will happen, “burning roots”, “red skin”, and “roots rot”, which will cause a serious yield reduction or even a period of non-harvest. For ginseng, it takes almost 30 years for the land to be suitable for replanting. Continuous cropping obstacles has become a pressing problem in ginseng cultivation that needs to be addressed. 

Plants can produce and release allelochemicals to interfere with the growth of the same species as well as interspecific plants. The allelopathic activity serves as an important medium between plant species in natural and managed ecosystems [4]. Autotoxicity is a type of autogenous toxic effect that affects plant growth through root secretion and other methods [5,6]. The autotoxicity of allelopathic substances is one of the main causes leading to the continuous cropping obstacles of ginseng. Currently, many allelochemicals from ginseng have been identified. Among them, ginsenosides are the most important active components in ginseng and are recognized as allelochemicals that cause autotoxicity in the research field of continuous cropping obstacles [7,8,9,10]. Therefore, the elucidation of the allelopathic autotoxicity mechanism of ginsenosides is of great significance in breaking the continuous cropping obstacle of ginseng. The literature reported that ginsenosides exert allelopathic activity through various pathways. Ginsenoside Rg_1_ induces the cell apoptosis of notoginseng root by the excessive accumulation of reactive oxygen species (ROS) [11]. The ginsenosides extracted from continuously cropped problem soil have an impact on the germination and growth of ginseng seedlings via significant changes in indicators such as superoxide dismutase (SOD) [12]. Ginsenosides Rg_1_, Rb_1_, Rh_1_, and their mixtures could disrupt the balance of fungal microbiota by stimulating potential soil-borne pathogens [13,14]. Under the stress of ginsenoside Ro, the accumulation of salicylic acid in ginseng hairy roots increases, while the accumulation of abscisic acid and jasmonic acid is inhibited [6]. Under the stress of ginsenoside Rg1, the genes related to cell membrane, cell wall breakdown, and ROS metabolism are upregulated [15]. Under the induction of ginsenoside Ro, ARF7 and EFMs are upregulated in the hormone signaling transduction pathway, while XTH23 and ZOX1 are downregulated [6]. It can be seen that some progress has been made in the elucidation of the allelopathic autotoxicity mechanism of ginsenosides. But there remains no clarification regarding the correlation mechanism between the polarity of ginsenosides and their allelopathic autotoxicity.

Herein, the apparent morphology, multiple proliferation, and physiological indexes of ginseng hairy roots under the stress of ginsenosides with different polarities were detected. By targeted phytohormone metabolomics, transcriptomics, and integrated analysis, the different accumulation levels of endogenous phytohormones and differential expression key genes were screened out. The related biological pathways were enriched. Further, the mechanism of the correlation between the polarity of ginsenosides and their allelopathic autotoxicity was explored. This investigation provided a reference for allelopathy research and soil amelioration of ginseng cultivation.

## 2. Results

### 2.1. Composition and Content of Ginsenosides in Different-Polarity Components

The composition and content of ginsenosides in different-polarity components were determined by UPLC-QQQ-MS (Table S1 and Figure S1). After data processing, ginsenosides were found mainly presented in high-polarity ginsenoside components (ZG50) and low-polarity ginsenoside components (ZG70), and were rarely detected in the lowest-polarity ginsenoside component (ZG100). Therefore, components ZG50 and ZG70 were selected to further study the correlation between the polarity and allelopathic autotoxicity of ginsenosides. 

### 2.2. Allelopathic Autotoxicity of Differen-Polarity Ginsenoside Components

#### 2.2.1. The Influence on Apparent Morphology of Ginseng Hairy Roots 

**Figure 1:**
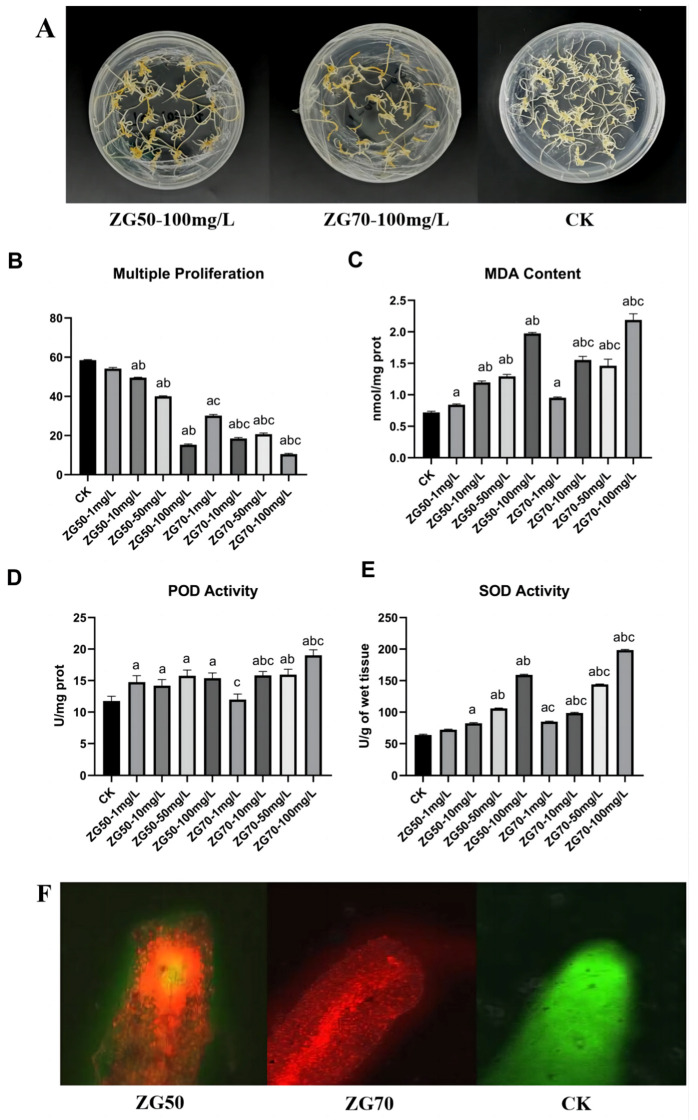
The stressing of different polarities of ginsenoside components on ginseng hairy roots: apparent morphology (**A**); the results of multiple proliferation (**B**); MDA content (**C**); POD activity (**D**); SOD activity (**E**); apical cell viability (**F**). Significant compared with CK group (a), compared of different concentrations within groups (b), and between ZG50 and ZG70 groups under the same concentration (c).

In control check (CK) group, ginseng hairy roots exhibited a light-yellow color with numerous branches and vigorous growth. Under different concentrations of components ZG50 and ZG70 (1 mg/L, 10 mg/L, 50 mg/L, and 100 mg/L), ginseng hairy roots presented different degrees of yellow to brown coloration, with decreased branches and sparse distribution. Among them, the groups with concentration at 100 mg/L showed the sparsest distribution and the darkest yellow color (Figure 1A). Meanwhile, the multiple proliferation results also showed that the 100 mg/L growth inhibition effect was the most significant (Figure 1B). 

Therefore, it could be seen that the allelochemicals induced by components ZG50 and ZG70 inhibited the growth of ginseng hairy roots and accelerated its senescence. However, the autotoxicity mechanism remained unknown. Therefore, further exploration was needed to investigate the correlation between the polarity of ginsenoside components and their allelochemical autotoxicity.

#### 2.2.2. The Influence on Apical Physiological Indexes of Ginseng Hairy Roots

According to the physiological index analysis (Figure 1C–E), there were significant differences in malondialdehyde (MDA) content and SOD activity of ginseng hair roots in ZG50 (100 mg/L) and ZG70 (100 mg/L) groups compared with CK. MDA was one of the major products of membrane lipid peroxidation; its accumulation could damage plant cell membranes and organelles [16]. SOD and peroxidase (POD) could balance the content of ROS in plants; the value changes showed that the balance of the antioxidant system in ginseng hairy root was broken by external stress. It indicated that different polar ginsenoside components drove the occurrence and development of allopathy by deepening the degree of the lipid peroxidation of ginseng hairy root membrane and by destroying the antioxidant system.

#### 2.2.3. The Influence of Ginseng Hairy Roots on Apical Cell Viability

ZG50 (100 mg/L), ZG70 (100 mg/L), and CK groups were selected for apical cell viability determination. The apical cells were observed using a confocal laser scanning microscope (Figure 1F). The live and dead cells were localized with a fluorescein diacetate-propidium iodide (FDA-PI) staining solution. After observation, the apical cells of the CK group showed green fluorescence and good cell viability. The cell viability of ZG70 and ZG50 groups were both damaged and produced abundant red fluorescence, showing that the growth of ginseng hairy roots was significantly inhibited. Among them, the cells of the ZG70 group were more severely damaged, which indicated that the polarity of the ginsenoside components is negatively correlated with their autotoxicity. It related to the fact that the ginsenosides in the ZG70 component were mainly of protopanaxadiol type.

### 2.3. Targeted Metabolomics Analysis of the Allelopathic Autotoxicity of Ginsenoside Components with Different Polarities

#### 2.3.1. Effects on Endogenous Phytohormone Accumulation and Metabolism in Ginseng Hairy Roots

**Figure 2:**
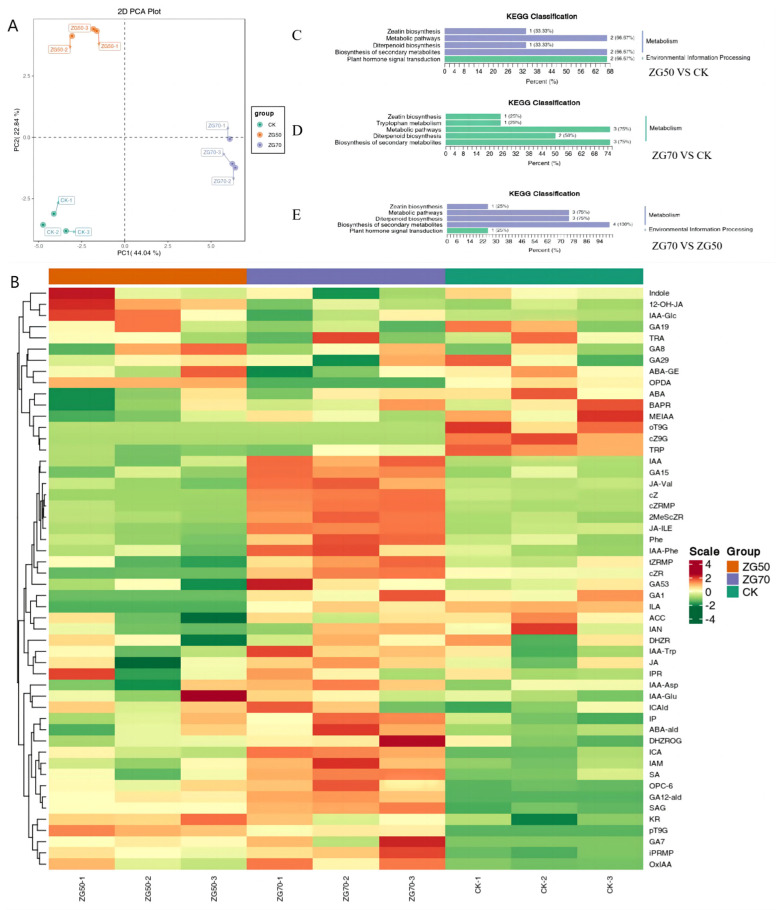
The PCA plot (**A**), HCA heatmap (**B**), and KEGG classification (**C**–**E**) of phytohormones and their metabolites.

Based on the LC-MS/MS platform, 109 endogenous phytohormones in nine categories were analyzed and the ion current chromatogram was extracted (Figure S2). The standard curves were established and the contents were calculated (Table S2). A total of 52 phytohormones and their metabolites were detected in ginseng hairy roots of ZG70, ZG50, and CK groups, which were completely quantified (Table S3). The PCA results showed significant differences among the ZG50, ZG70, and CK groups, with good repeatability within the groups (Figure 2A). 

Through HCA analysis, the accumulation of phytohormones and their metabolites among different groups is shown in the heatmap (Figure 2B). The three repeated samples within each group showed similar trends of the accumulation of the phytohormones’ metabolites. Among the groups, the metabolism of endogenous phytohormones in ginseng hairy roots of ZG70 was significantly different. It could be deduced that under the stress of ginsenosides in the ZG70 component, the endogenous phytohormones in ginseng hairy roots were intervened more significantly. 

To accurately identify differential phytohormone metabolism, fold change ≥ 2 and fold change ≤ 0.5 were used to screen significantly different endogenous phytohormone metabolites between two groups using FC analysis. A total of nine different phytohormone metabolites were screened out in the ZG50 vs. CK groups, including 4-[[(9-beta-D-glucopyranosyl-9H-purin-6-yl)amino]methyl]phenol (pT9G), gibberellin A12 aldehyde (GA12-ald), gibberellin A7 (GA7), and 3-oxo-2-(2-(Z)-pentenyl)cyclopentane-1-hexanoic acid (OPC-6) with increased accumulation, while indole-3-lactic acid (ILA), cis-zeatin (cZ), cis-zeatin-9-glucoside (cZ9G), ortho-topolin-9-glucoside (oT9G), and gibberellin A1 (GA1) were screened with decreased accumulation. In the ZG70 vs. CK group, 13 different phytohormone metabolites were identified. The accumulations of indole-3-carboxylic acid (ICA), indole-3-acetamid (IAM), cis-zeatin riboside monophosphate (cZRMP), cZ, pT9G, 2-methylthio-cis-zeatin (2MeScZ), GA12-ald, GA7, OPC-6, and salicylic acid 2-O-β-glucoside (SAG) increased, whereas the cZ9G, oT9G, and cis(+)-12-oxophytodienoic acid (OPDA) decreased. For the ZG70 vs. ZG50 group, eight different phytohormone metabolites, which included an accumulation of indole-3-lactic acid (ILA), cZRMP, cZ, 2MeScZ, GA1, n-[(-)-jasmonoyl]-(L)-valine (JA-Val), and jasmonoyl-L-isoleucine (JA-ILE), increased, while OPDA decreased. The significantly different phytohormones screened out were auxin, cytokinin, gibberellin, and jasmonates (JAs), all of which played an important role in the growth of plants.

#### 2.3.2. Metabolic Pathway Analysis of Differential Accumulation of Endogenous Phytohormones in Ginseng Hairy Roots

The metabolic pathways with significantly differential endogenous phytohormones were enriched using the KEGG database (Figure 2C–E). In the group ZG50 vs. CK, the biosynthesis of zeatin, diterpenoid, and secondary metabolites as well as metabolic pathways and phytohormone signal transduction were enriched. In the group ZG70 vs. CK, the biosynthesis of zeatin and diterpenoid, metabolic pathways, and tryptophan metabolism were enriched. In the group ZG70 vs. ZG50, the biosynthesis of zeatin, diterpenoid, and secondary metabolites as well as metabolic pathways and phytohormone signal transduction were also enriched.

### 2.4. Transcriptomics Analysis of the Allelopathic Autotoxicity of Ginsenoside Components with Different Polarities

#### 2.4.1. Transcript Splicing, Gene Annotation, and Differential Expression Gene Screening

To explore the molecular mechanisms of the correlation between polarity and autotoxicity of ginsenoside components, transcriptome sequencing of ginseng hairy roots of ZG50, ZG70, and CK groups was performed. The values of RNA integrity ranged from 6 to 7.6. The RNA concentration ranged from 257 ng/μL to 1004 ng/μL. The hairy root samples of each group passed the RNA quality inspection and met the requirements of subsequent experiments. Based on the sequencing by synthesis (SBS) technology, the prepared cDNA libraries were sequenced by the Illumina high-throughput sequencing platform. The high-quality transcriptome sequencing data were obtained (Table 1). Transcript and Unigene obtained 214,575 and 122,081 sequences, respectively. The average sequence lengths were 1060 and 1283; N50 was 1689 and 1873; N90 was 445 and 580, respectively. The total bases were 227,410,317 and 156,674,968.

**Figure 3:**
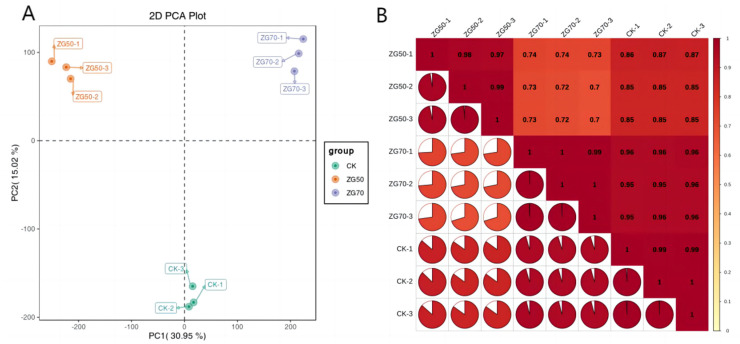
The results of transcriptomics analysis. (**A**) PCA plot. (**B**) Sample correlation plot.

To recognize the key factors of the transcriptome data, PCA was used to demonstrate the changes in ZG50, ZG70, and CK (Figure 3A). The PCA results showed that the first two principal components (PC1, PC2) could clearly distinguish these three groups. In addition, Pearson’s correlation coefficient was used as an assessment indicator of biological repetition correlation. The closer the absolute value of r is to 1, the stronger the similarity of duplicate samples. From the sample correlation plot (Figure 3B), the similarity between biological replicates within the same group was high, which indicated good reproducibility of the ZG50, ZG70, and CK samples.

The screening conditions for differential genes in this study were |log2Fold Change| ≥ 1 with FDR < 0.05. A total of 5442 differential genes were screened out in the ZG50 vs. CK group, of which 3890 genes were downregulated and 1552 genes were upregulated. In the group ZG70 vs. CK, 7380 differential genes were screened out, of which 3785 genes were downregulated and 3595 genes were upregulated. A total of 13,847 differential genes were screened out in the ZG70 vs. ZG50 group, of which 6038 genes were downregulated and 7809 genes were upregulated. 

#### 2.4.2. The Functional Annotation and Enrichment of Differential Genes 

To further explore the biological function of the differentially expressed genes, GO, KEGG, and KOG enrichment analyses were performed.

##### KEGG Enrichment Analysis

For KEGG enrichment analysis (Figure S3), 135 pathways associated with the ZG50 vs. CK group’s differential genes were mainly enriched through protein processing in endoplasmic reticulum, oxidative phosphorylation, protein export, RNA degradation, and the metabolism of glutathione, arachidonic acid, porphyrin, vitamin B6, and glycerolipid. A total of 140 metabolic pathways were associated with the ZG70 vs. CK group’s differential genes (Figure S4). They were mainly enriched in the biosynthesis of secondary metabolites, phenolylpropanoid, unsaturated fatty acids, varotenoid, stilbenoid, diarylheptanoid and gingerol, metabolic pathways, metabolism of cyanoamino acid, starch, and sucrose, and phytohormone signal transduction. 

**Figure 4:**
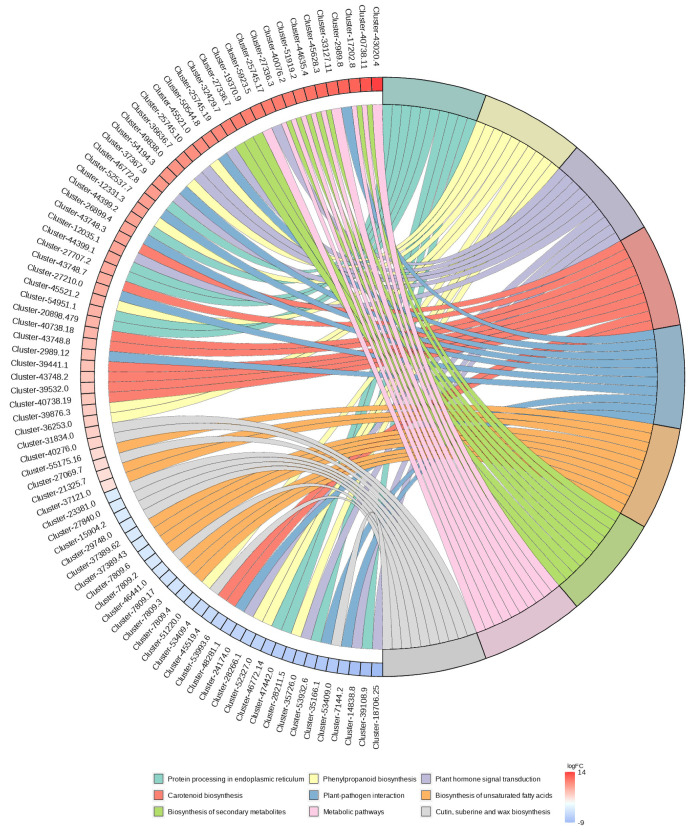
Enrichment chord of KEGG enrichment of ZG70 vs. ZG50 group.

As shown in Figure 4, for the ZG70 vs. ZG50 group, 145 metabolic pathways associated with differential genes were mainly involved in protein processing in endoplasmic reticulum, phytohormone signal transduction, biosynthesis of phenylpropanoid, carotenoid, unsaturated fatty acids, cutin, suberine, wax and secondary metabolites, metabolic pathways, plant–pathogen interaction, etc. The protein processing in endoplasmic reticulum, phytohormone signal transduction, and phenylpropanoid biosynthesis pathways was more significantly differently expressed in the ZG70 and ZG50 groups, which drove the occurrence of autotoxicity.

##### GO Enrichment Analysis

GO enrichment analysis showed that (Figure S5) the ZG50 vs. CK group enriched 3209, 3438, and 3371 differential genes with regard to biological process, cellular component, and molecular function, respectively. Among them, 92 downregulated genes appeared (2.87%) in protein refolding. Sixty-two genes were downregulated and seven genes were upregulated in response to topologically incorrect protein, and differential genes accounted for 2.15%. The 68 genes that changed in cellular response to topologically incorrect protein, including 62 downregulated and 6 upregulated proteins, were ranked third. In addition, 53 genes were downregulated at the endoplasmic reticulum lumen under the cellular component category. In the molecular function category, 78 genes were downregulated in the heat shock protein binding function, and 40 genes were enriched for the misfolded protein binding function. It suggested that ZG50 induced allelopathy through the negative regulation of the correct folding of the endoplasmic reticulum’s transport protein peptide. Additionally, ZG50 prevented the protein from smoothly entering the Golgi apparatus for further glycosylation and from performing other modifications. An excessive accumulation of faulty proteins or unfolded proteins in the endoplasmic reticulum triggered stress mechanisms. Furthermore, the expression of related genes triggered the initiation of the apoptosis program, which was manifested by an accelerated aging of ginseng hairy roots.

The ZG70 vs. CK group enriched 4380, 4691, and 4632 differential genes under the biological process, cellular component, and molecular function categories, respectively (Figure S6). ZG70 triggered more differentially expressed genes than ZG50. Under the biological process category, phenylpropanoid biosynthetic process enriched 51 downregulated genes and 14 upregulated genes. The cellular responses to decreased and unchanged oxygen levels both enriched 45 downregulated genes and 20 upregulated genes. Under the cellular component category, nucleosome enriched 29 downregulated genes and three upregulated genes. The anchored component of the plasma membrane enriched 29 downregulated genes and 17 upregulated genes. Under the molecular function category, dioxygenase activity was the most enriched function for the differential genes, including 28 downregulated genes and 36 upregulated genes. Symporter activity followed, which enriched 30 downregulated genes and 16 upregulated genes. It was demonstrated that ZG70 could drive the occurrence and development of autotoxicity by regulating phenylpropanoid biosynthesis, cellular reactive oxygen species level, and transporter activity.

**Figure 5:**
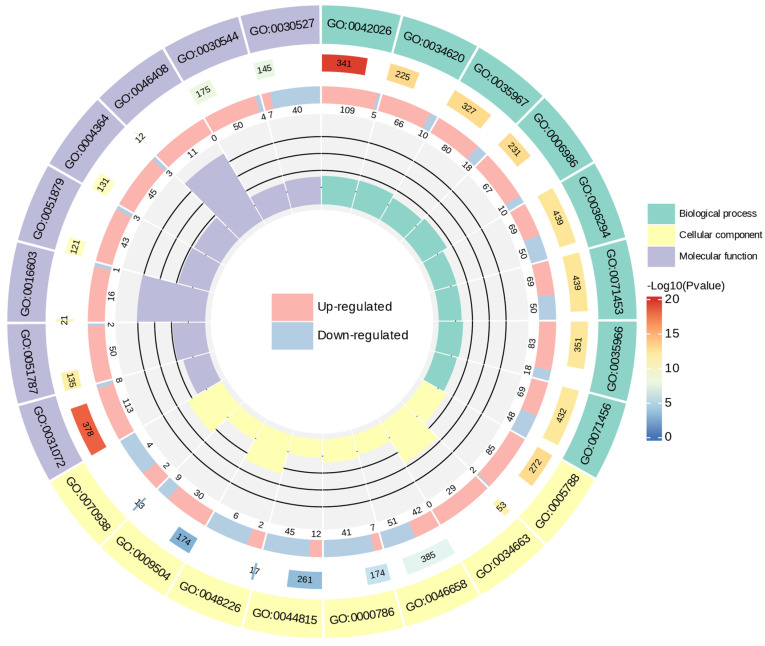
Circle plot of GO enrichment of ZG70 vs. ZG50 group.

The ZG70 vs. ZG50 group enriched 8325, 8902, and 8748 differential genes in the biological process, cellular component, and molecular function categories, respectively (Figure 5). The biological changes caused by ZG50 and ZG70 treatments were different and the ginsenoside components with different polarities induced different allelopathic mechanisms. Under the biological process category, cellular responses to decreased oxygen levels, oxygen levels, and hypoxia enriched more differential genes, with upregulated genes more than the downregulated ones. Compared to ZG50, the ZG70 group positively regulated the cellular stress response to hypoxia. It indicated that under the ZG70 treatment, the cellular hypoxia condition was even more severe. In the cellular component category, the anchored components of plasma membrane and endoplasmic reticulum lumen enriched 93 and 87 differential genes, respectively, whereby the latter contained 85 upregulated genes. Under the molecular function category, heat shock protein binding enriched the most differential genes, including eight downregulated genes and 113 upregulated genes. The POD activity enriched 38 downregulated genes and 42 upregulated genes. Compared with ZG50, increasingly severe faulty folding occurred in the protein expression of ginseng hairy roots under ZG70 stress. The ginsenosides with low polarity made the ginseng hairy roots positively regulated through the expression of heat shock proteins, thus enabling the abnormal protein recovery to resume normal function.

##### KOG Enrichment Analysis

The KOG annotation results also confirmed the results of KEGG and GO enrichment analyses. ZG50 vs. CK, ZG70 vs. CK, and ZG70 vs. ZG50 were mainly annotated to post-translational modification, protein turnover, chaperones, general function prediction, and phytohormone signal transduction mechanisms.

#### 2.4.3. Validation of the Key Genes by qPCR

To verify the accuracy of transcriptomic data, real-time qPCR analysis was performed on the 12 genes associated with phytohormone signal transduction, diterpenoid biosynthesis, tryptophan metabolism, and zeatin biosynthesis pathways. The results of the transcriptome showed similarity to those of the qPCR and consistent expression trends, validating the reliability of the RNA-seq results (Table S4).

### 2.5. Integrated Metabolomic and Transcriptomic Analysis of the Correlation between Polarity and Allelopathic Autotoxicity of Ginsenosides

**Figure 6:**
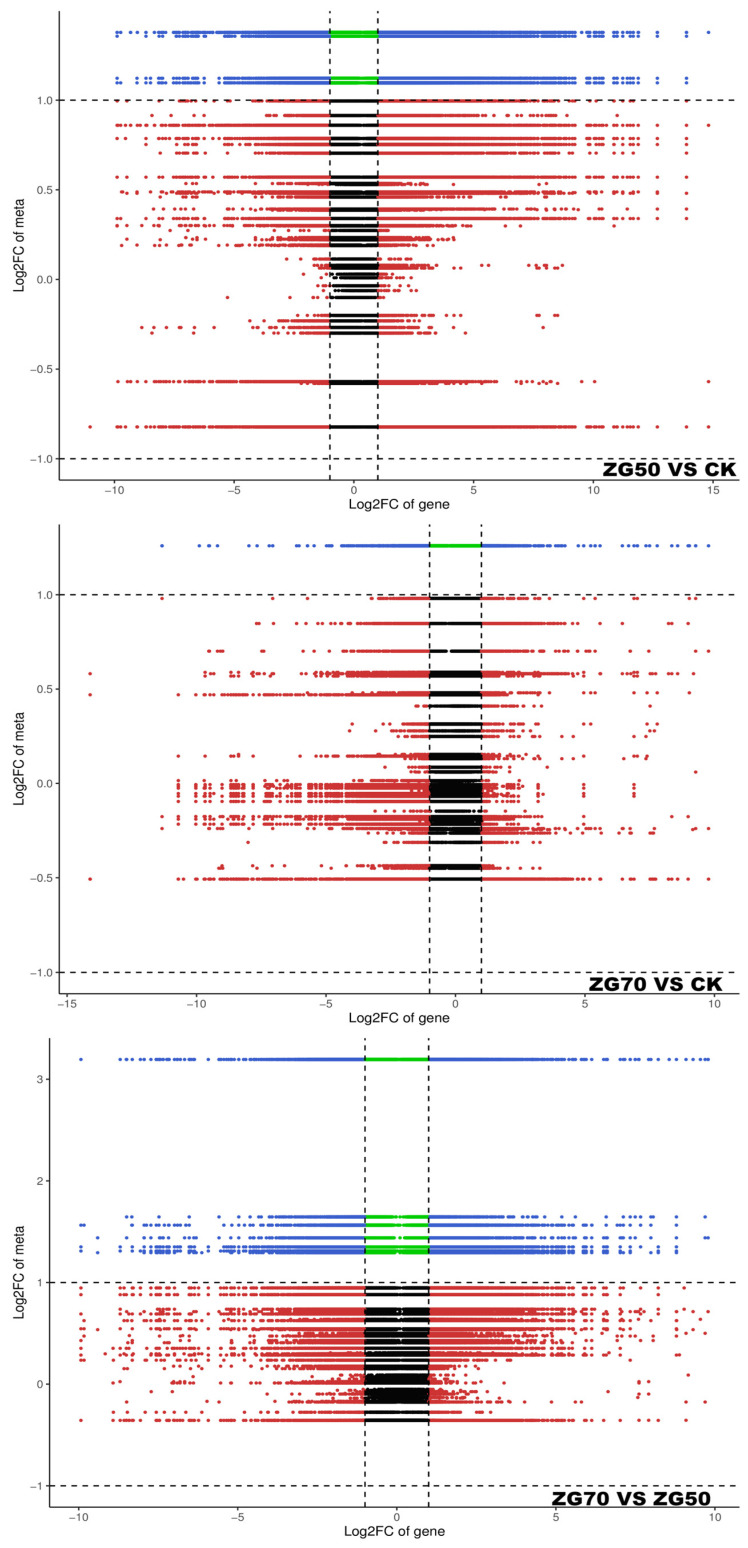
Nine-quadrant plots of correlation analysis of differential genes and phytohormones for ZG50 vs. CK, ZG70 vs. CK, and ZG70 vs. ZG50 groups. With a black dashed line, from left to right, from top to bottom, successively divided into 1–9 quadrants. The 5 quadrant represents both non-differentially expressed genes and metabolites. The 3 and 7 quadrants represent genes consistent with the differential expression patterns of the metabolites, namely that the expression changes of the metabolites may be positively regulated by the genes. The 1 and 9 quadrants represent genes with opposite differential expression patterns with metabolites, namely that the expression changes of metabolites may be negatively regulated by the genes. The 2, 4, 6, and 8 quadrants represent metabolites with no correlation with genes changes.

**Figure 7:**
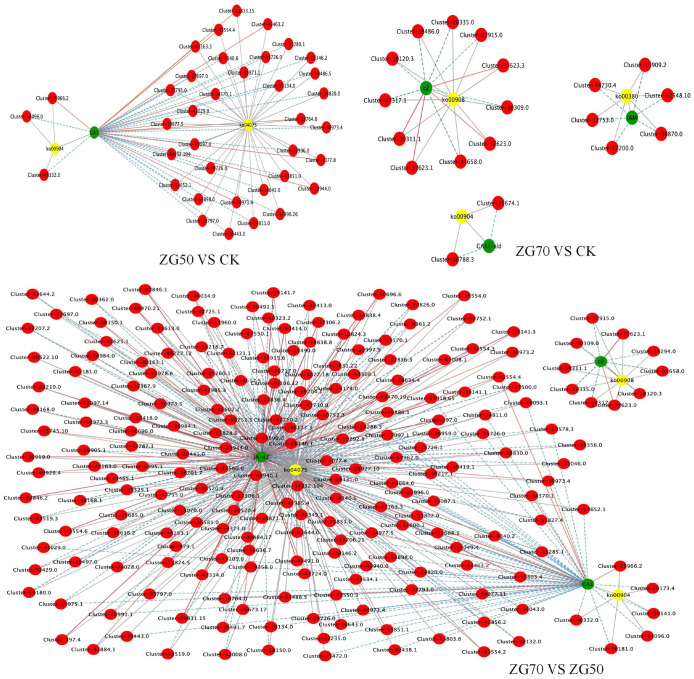
Correlation network diagrams of correlation analysis of differential genes and phytohormones for ZG50 vs. CK, ZG70 vs. CK, and ZG70 vs. ZG50 groups.

To further explore the molecular mechanism of the occurrence and development of allelopathy driving by different polar ginsenoside components, the relationship between significantly different phytohormone metabolites and expressed genes of ZG50 vs. CK, ZG70 vs. CK, and ZG70 vs. ZG50 groups was investigated. The integrated analysis was performed based on the differential phytohormone metabolites and genes screened out as shown below (Table 2). The differential genes and phytohormones with a Pearson’s correlation coefficient greater than 0.80 and *p* value less than 0.05 in each pathway were selected for correlation analysis, which were displayed in nine-quadrant plots (Figure 6). According to KEGG pathway maps, metabolic pathways and the biosynthesis of secondary metabolites were overview pathways, and the metabolites and genes could not be viewed. Therefore, the correlation networks of the other pathways are shown in Figure 7.

**Figure 8:**
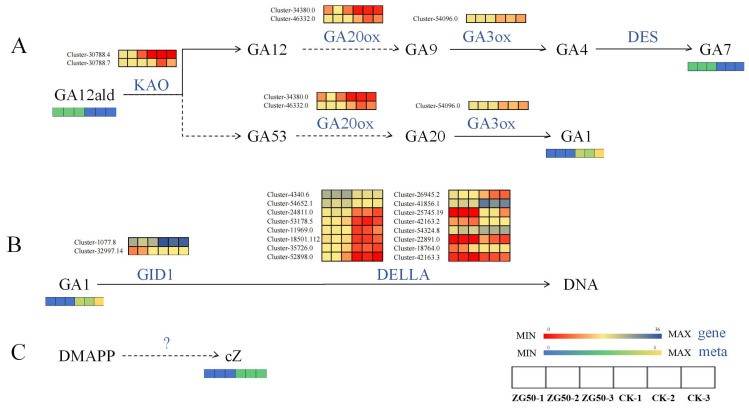
The pathway heatmaps of differential genes and phytohormones for ZG50 vs. CK group. (**A**) Diterpenoid biosynthesis pathway. (**B**) Phytohormone signal transduction pathway. (**C**) Zeatin biosynthesis pathway.

ZG50 vs. CK: In the diterpenoid biosynthesis pathway (Figure 8A), the expression genes of ent-kaurenoic acid monooxygenase (KAO) in terpenoid skeleton synthesis were upregulated. The accumulation of GA12-ard was increased by the stereotactic hydroxylation of C-7β and carbon–carbon double bond migration at positions C7 and C8 of the B ring [17]. The gibberellin A53 aldehyde (GA53-ard) was also indirectly generated. Then, GA12-ard and GA53-ard were oxidized to gibberellin A12 (GA12) and gibberellin A53 (GA53), which were converted to gibberellin A9 aldehyde (GA9) and gibberellin A20 (GA20), respectively. This process depended on the 2-odd family gibberellin-44 dioxygenase (GA20ox). The GA20ox cleaved at C-20 with the formation of the 19,10-γ-lactone characteristic of C19-GAs. In the reaction sequence, the C-20 methyl was oxidized to the alcohol and then to the aldehyde [18]. The genes Cluster-34380.0 and Cluster-46332.0 that were related to GA20ox were upregulated. In the final step of GA biosynthesis, GA9 and GA20 were 3β-hydroxylated by gibberellin 3β-dioxygenase (GA3ox) into gibberellin A4 (GA4) and GA1, respectively. The expression of the GA3ox genes was upregulated, which negatively regulated the accumulation of GA1. Interestingly, there was no change in the final product GA4, while the intermediate byproduct GA7 showed an upregulation upon overexpression of GA3ox. It was similar with that reported in the *Marah macrocarpu* [19]. Under the phytohormone signal transduction pathway (Figure 8B), the decrease in GA1 causes the downregulation of the gibberellin receptor GID1 and its genes (Cluster-1077.8 and Cluster-32997.14), which in turn causes a strong change in the expression of DELLA protein genes. The DELLA protein was a suppressor during gibberellin signaling [20]. At a low concentration of gibberellin, DELLA protein bound to downstream key regulatory factors, and the ubiquitination modification of DELLA protein was inhibited. Therefore, the signal transduction of gibberellin was inhibited and it negatively regulated the growth of the plant stem. In the zeatin biosynthesis pathway (Figure 8C), the accumulation of cZ decreased, but the genes which regulated this process remained unknown. Zeatin synthesis included the tRNA pathway and the AMP pathway, while cZ works mainly through the tRNA pathway [21,22].

**Figure 9:**
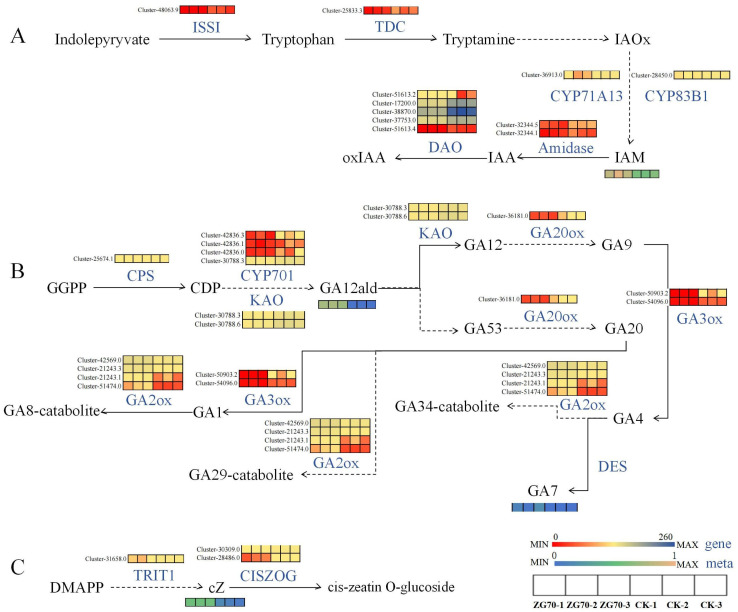
The pathway heatmaps of differential genes and differential metabolites for ZG70 vs. CK group. (**A**) Tryptophan metabolism pathway. (**B**) Diterpenoid biosynthesis pathway. (**C**) Zeatin biosynthesis pathway.

ZG70 vs. CK: Under the tryptophan metabolism pathway (Figure 9A), indole pyruvate was converted to tryptophan by aromatic aminotransferase (ISS1) (downregulated in Cluster-48063.9). Tryptophan was then decarboxylated to tryptamine by L-tryptophan decarboxylase (TDC) (downregulated in Cluster-25833.3). Tryptamine underwent a series of reactions to generate intermediate indole 3-acetaldoxime (IAOx). IAOx was then converted to indole 3-acetonitrile (IAN) by indoleacetaldoxime dehydratase (CYP71A13, downregulated in Cluster-36913.0) and aromatic aldoxime N-monooxygenase (CYP83B1, downregulated in Cluster-28450.0), and led to an increased accumulation of the inactive auxin precursor 3-indoleacetamide (IAM). Under the diterpenoid biosynthesis pathway (Figure 9B), there was a differential expression of genes at the initiation stage of diterpenoid skeleton synthesis. GAs were formed from trans-geranylgeranyl diphosphate (GGPP) [23], which was performed via entcopalyl diphosphate synthase (CPS) cyclization to obtain entcopalyl diphosphate (CDP) [24]. The CPS gene Cluster-25674.1 was downregulated, which was the first key enzyme gene to enter the gibberellin biosynthetic pathway. The expression of the ent-kaurene oxidase (CYP701)-related genes and the KAO genes decreased, while the level of GA12-ard increased. This finding suggests that KAO indirectly regulated GA12-ard, and not directly. The expression of genes related to GA20ox (Cluster-36181.0) was downregulated. At the same time, the expression of genes related to GA3ox (Cluster-50903.2 and Cluster-54096.0) were downregulated, but no difference in GA1 accumulation was observed, indicating that the expression patterns of GA3ox and GA1 were not correlated. It was similar with the GA3ox gene regulation of the ZG50 group, where no changes occurred in GA4, while the intermediate byproduct GA7 had increased. Under the zeatin biosynthesis pathway (Figure 9C), the related gene Cluster-31658.0 in the tRNA dimethylallyl transferase (TRIT1) pathway was downregulated, which negatively regulated cis-zeatin to increase its accumulation. Subsequently, the cis-zeatin O-glucosyltransferase (cis ZOG) responsible for metabolizing cZ increased and was negatively regulated by genes Cluster-30309.0 and Cluster-28486.0.

**Figure 10:**
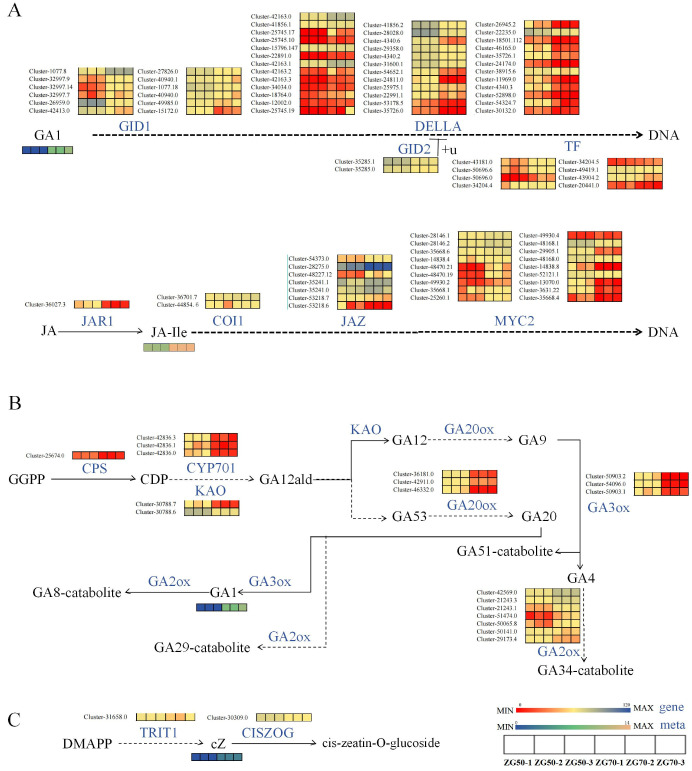
The pathway heatmaps of differential genes and differential metabolites for ZG70 vs. ZG50 group. (**A**) Hormone signal transduction pathway. (**B**) Diterpenoid biosynthesis pathway. (**C**) Zeatin biosynthesis pathway.

ZG70 vs. ZG50: The increased accumulation of GA1 under the phytohormone signal transduction pathway (Figure 10A) led to more significant changes in the expression of GID1, DELLA, and TF genes. Meanwhile, the key gene Cluster-36027.3 of jasmonic acid-amino synthetase (JAR1) [25] was downregulated, which negatively regulated the synthesis of JA-ILE and led to an increased accumulation of JA-ILE. JA-ILE is the main active form of Jas and is perceived by the coronatine-insensitive protein 1 (COI-1) receptor (upregulated expression of genes Cluster-36701.7 and Cluster-44854.6). Jasmonate ZIM domain-containing protein (JAZ) (five upregulated genes, two downregulated genes) was recruited for ubiquitination and degradation through the 26S proteasome [26]. Therefore, the inhibition on transcription factor MYC2 (10 upregulated genes, eight downregulated genes) was alleviated and allowed the expression of JA-responsive genes. The diterpenoid biosynthesis pathway (Figure 10B) underwent similar changes with those observed in ZG70 vs. CK, but there was no significant accumulation of the byproduct GA7. The expression relationships between highly significant phytohormone metabolites and key genes in the zeatin biosynthesis pathway (Figure 10C) were basically consistent with those of ZG70 vs. CK.

## 3. Discussion

### 3.1. Innovative Study on Allelopathic Autotoxicity of Different Polarities of Ginsenoside Components 

In this study, the ginsenosides were separated based on different polarities, and two polar fractions with relatively higher ginsenoside distributions (ZG50 and ZG70) were selected for further research. The correlation between the polarity of ginsenoside fractions and their allelopathic autotoxicity was explored. The results showed that the allelopathic autotoxicity of ZG50 and ZG70 inhibited the growth and accelerated the senescence of ginseng hairy roots. The low polarity fraction ZG70 presented more severe allelopathy. The composition of ginsenoside components in ZG70 and ZG50 was different. The ZG50 component which had the majority of ginsenosides contained a hydroxy at C-6 and/or formed glycosides, while the ginsenosides in the ZG70 component were without substituents at C-6. This might be one of the reasons for the stronger allelopathic effect of the component with low polarity, which was innovative compared to reported ones. Current research has mainly focused on the allelopathic activities of total ginsenosides and individual ginsenosides. Generally, ginsenosides accumulated in rhizosphere soils through root secretion and/or root decomposition ways, thereby inhibiting seedling emergence and growth [15]. Ginsenosides Rb_1_ and Re at high concentration could induce reduced proliferation of ginseng cells and decreased branching of adventitious roots [27]. High concentration of total ginsenosides had an inhibitory effect on the growth of American ginseng seedlings [28]. Different concentrations of ginsenoside Rd had different allelopathic effects on the germination and seedling growth of ginseng seeds [8].

### 3.2. Exploring the Possible Mechanism of the Relationship between the Polarity and Allelopathic Autotoxicity of Ginsenoside Components

#### 3.2.1. Reduce the Cell Vitality of Ginseng Roots

FDA is a non-fluorescent hydrophobic derivative of fluorescein. FDA can actively diffuse into cell membranes and undergo intracellular esterase-mediated hydrolysis of the diacetate group to generate a strong fluorescent product, fluorescein. Therefore, fluorescein accumulates in live cells with intact membranes and emits a green fluorescence. PI is a dead cell marker that cannot penetrate the cell membrane and is excluded by live cells but can pass through damaged cell membranes, stain the nucleus, and produce red fluorescence [29,30]. Through a confocal laser scanning microscope observation of the apical cells, it was found that both ZG50 and ZG70 groups presented large areas of red fluorescence compared with CK, with a decrease in cell vitality. In contrast, ZG70 cells showed a higher degree of damage than ZG50 cells, which indicated that the polarity of ginsenoside components was correlated with the strength of autotoxicity. One of the mechanisms by which ZG50 and ZG70 induced autotoxicity was to reduce cell vitality and drive cell apoptosis. This result was supported by the conclusion in the literature; after treatment with 0.5 mg/L ginsenoside Ro, the apical cell vitality of ginseng hairy root was significantly reduced [6].

#### 3.2.2. Differential Expression of Key Genes Induced Phytohormones and Their Derivatives

##### Regulate the Expression of Genes of Key Enzymes in the cZ Synthesis Pathway

Cytokins play a crucial role in various aspects of plant growth, such as promoting cell proliferation, regulating leaf senescence, and responding to biotic or abiotic stress [31]. cZ is one of cytokinin, a stereoisomer of trans-zeatin (tZ). The cZ was once considered without biological activity, but subsequent research shows that this is not the case [21]. Currently, much of the functions of cZ remain unknown [21,32]. Research found that under inorganic phosphorus (Pi) starvation conditions, Arabidopsis thaliana would increase the ratio of cZ/tZ to maintain its response to the cytokinin signaling pathway. It demonstrated that cZ was a hormone that responded to Pi deficiency and could promote elongation of roots and hairy roots, and increased the ability of the roots system to absorb Pi from the environment [32]. In one study, the gene of cis-zeatin-O-glucosyltransferase (cisZOGT1) played an important role in the phenotypic characteristics of wheat [33]. Coincidentally, cisZOGT1 was also found to be isolated from corn, which indicated that cZ and its derivatives might be even more important in the homeostasis of cytokinin than currently recognized [34]. 

In this study, significant differences in cZ accumulation were observed between ZG50 vs. CK, ZG70 vs. CK, and ZG70 vs. ZG50 groups. The autotoxicity mechanism of ZG70 was achieved through regulating the gene expression of TRIT1 and cisZOG, increasing the content of cZ, reducing the overall activity of cytokinin in ginseng hairy roots, and resulting in growth inhibition. It supported the conclusion in the literature that plants under biotic and abiotic stress conditions would increase cZ via stress induction to maintain minimum cytokinin activity, and thus form self-protection [35,36]. It is worth mentioning that the stress of ZG50 reduced the accumulation of cZ by a process which involved unknown rate-limiting or -blocking enzymes in the tRNA pathway from prenyl-tRNA to cZ, which remains to be further explored [37].

##### Regulate the Expression of Genes of Key Enzymes in the Gibberellin Signal Transduction Pathway

Gibberellins (GAs), a group of ubiquitous diterpenoid phytohormones, mainly promoted plant growth and development through the increase in cell number and cell elongation, and played an important role in plant stress [20]. Under the stress of ZG50 and ZG70, significant changes were observed in the levels of GA1, GA7, and GA12-ald in hairy roots of ginseng. One of the mechanisms of the induction of allelopathy by ZG50 and ZG70 was through the regulation of the gene expression of key enzymes in the GA synthesis pathway, including GID1, KAO, GA20ox, and GA3ox, which resulted in a decrease in highly bioactive GA1 and thereby inhibiting the growth of ginseng hairy roots.

##### Regulate the Expression of Genes of Key Enzymes in the Pathways of Indole-3-acetic acid (IAA) Synthesis and Oxidation Inactivation

Auxin is an endogenous phytohormone containing an unsaturated aromatic ring and an acetate side chain, which can drive plant growth and morphogenesis [38,39,40]. IAA is a natural active auxin that is mainly synthesized through the tryptophan-dependent pathway. The tryptophan-dependent pathway included the indole-3-pyruvate pathway, indole-3-acetamide pathway, and indole-3-acetaldoxime pathway [41,42]. Plants had strict control over the level and distribution of IAA through synthesis, inactivation, and transport pathways. Some precursors and modified forms of auxin, which had been used to regulate the homeostasis of auxin, were identified; they included naturally generated inactive auxin precursors such as IAM [43], indole-3-acetaldoxime (IAOx) [44], and amino acid derivatives such as indoleacetyl glutamic acid (IAA-Glu) [45].

Under the stress of ZG70, ginseng hairy roots showed significant differences in IAM, the inactive precursor in the auxin synthesis pathway. The literature indicates that IAM could be converted into active IAA by amidase [46]. There was indeed a change in the amidase gene in this study, but there was no significant difference in IAA. The reason might be that it initiated the IAA oxidative inactivation pathway. According to the KEGG pathway, under the stress of ZG70, the genes of the DAO underwent significant changes. DAO is a key marker enzyme that regulates the deactivation of auxin, which plays an important role in maintaining auxin homeostasis. We could believe that active IAA might be deactivated through the GH3-ILR1-DAO pathway and the irreversibly generated oxindole-3-acetic acid (oxIAA), thereby terminating auxin signaling transmission [41,47,48,49]. One of the allelopathy mechanisms which was induced by ZG70 was achieved through regulating key genes ISS1, TDC, CYP71A13, and CYP83B1 to cause the differential accumulation of IAM, while regulating the key gene DAO to initiate the IAA oxidative deactivation pathway and then to disrupt the auxin signaling transduction and to inhibit the growth of ginseng hairy roots.

##### Regulate the Expression of Genes of Key Enzymes in JA Signaling Transduction Pathway

In the process of adaptation to terrestrial environments, plants have evolved approximately 500 types of JAs derived from polyunsaturated fatty acids. The JAs regulate the ecological interaction between plants, the biotic and abiotic environments, and the coordinated growth and development of plants [50]. The function of JAs depends on its signaling transduction module, which consists of the amino acid-coupled bioactive compound JA-ILE, the JA receptor COI-1 protein, the repressor protein JAZ, and transcription factors MYCs, MYBs, MED25, etc. [51,52].

Compared with ZG50, significant differences in JA-ILE accumulation were observed in ginseng hairy roots responding to the stress of ZG70. This indicated that JA-ILE was a key response factor to ZG70 stress. One of the allelopathy mechanisms induced by ZG70 was the regulation of the gene expression of key enzymes in the JA signaling pathway, including JAR1, COI-1, JAZ, and MYC2, thereby enhancing the stress response.

In this study, the correlation between the polarity of ginsenoside components and their allelopathic autotoxicity was explored. Ginsenoside components with low polarity presented stronger allelopathic autotoxicity. The molecular mechanism was also explored, and provided a new theoretical basis and direction for solving the continuous cropping obstacle of ginseng. There were also limitations to this study. Under the stress of ginsenosides with different polarities, the accumulation of cZ in the hairy roots of ginseng showed an opposite trend. It was deduced that there might be an unknown rate-limiting or -blocking enzyme in the tRNA pathway, which needed to be further explored. The DELLA protein family is widely present in Arabidopsis, rice, and other plants; its structural domains are highly conserved. The DELLA protein SLR1 was discovered in rice [53]. Five DELLA proteins were identified in *Arabidopsis thaliana*: RGA [54], GAI [55], RGL1 [56], RGL2 [57], and RGL3 [58]. Five DELLA proteins were identified in *Brassica oleracea* var. *capitate* [59]. However, up to now, no DELLA protein has been identified in the species of ginseng. In this study, the key gene of the DELLA protein was found to be significantly changed, which indicated that it was highly related to the allelopathic autotoxicity of ginsenosides. cZ, IAM, GA1, and JA-ILE were identified as the key response factors to the stress of ginsenoside components of different polarity in the hairy roots of ginseng. However, the results are still inconclusive and need to be further explored.

## 4. Materials and Methods

### 4.1. Plant Material

Ginseng roots (6-year-old garden ginseng, variety named “Fuxing No. 2”) were collected from Songjianghe Town, Fusong County, Baishan City, Jilin Province, China (127°48′ E, 42°18′ N), and authenticated by Professor Changbao Chen from Changchun University of Traditional Chinese Medicine.

The hairy roots have the characteristics of fast propagation and high genetic stability. To ensure the uniformity of the experimental material, the hairy roots used in this study came from the same root hairs of a single ginseng.

### 4.2. Preparation and Determination of Ginsenoside Components with Different Polarities

#### 4.2.1. Preparation of Ginsenoside Fractions using Medium-pressure Liquid Chromatography

Fresh ginseng roots were dried, powdered, and passed through a 200-mesh sieve. An amount of 10 g of powder was weighed and added to 200 mL of 70% methanol. Ultrasonic extraction was performed for 1 h, and the supernatant was collected. The extraction was repeated and the supernatant was collected and combined. The combined supernatant was centrifuged at 9000 rpm for 10 min. The filtrate was concentrated under reduced pressure and then extracted with water-saturated butanol. The butanol layer extract was then evaporated to dryness at 45 °C, and the final solution was made up to 10 mL with 70% methanol, obtaining the ginsenoside extract solution.

Medium-pressure liquid chromatography (Flash Isolera one, Biotage Sweden AB Inc., Uppsala, Sweden) coupled with preparation column (120 g Biotage^®^ Sfar C18) was conducted. The mobile phase consisted of water (A) and methanol (B) with a flow rate of 50 mL/min and a maximum column volume of 164 mL (1CV). The gradient separation program was as follows: A/B 40% for 4CV, initial waste; A/B 50% for 12CV, full collection; A/B 70% for 12CV, full collection; and A/B 100% for 16CV, full collection. Three different polarities of ginsenoside fractions were collected, namely 50% (ZG50), 70% (ZG70), and 100% (ZG100), which were vacuum-concentrated to 10 mL, respectively. A total volume of 1 mL of different-polarity ginsenoside fractions (ZG50, ZG70, and ZG100) was taken, filtered through a 0.22 μm Millipore filter, and stored at 4 °C for determination.

#### 4.2.2. UPLC-QQQ-MS Determination of Ginsenoside Components with Different Polarities

A TTSQ Endura UPLC-QQQ-MS coupled with Syncronis C18 (100 mm × 2.1 mm, 1.7 μm) (Thermo Scientific Inc., Waltham, MA, USA) was used. The specific protocol was performed as previously described [60]. Fifteen kinds of ginsenoside reference substances (Rg_1_, Rf, Rb_1_, Rc, Ro, Rb_2_, Rd, Re, Rg_2_, Rh_1_, NotoR_2_, Rb_3_, F_2_, and Rg_3_) were weighed, the standard curves were established, and the composition and contents of the ginsenosides in different-polarity components were determined.

### 4.3. Allelopathic Autotoxicity Validation

#### 4.3.1. Culture of Ginseng Hairy Roots

Different polarities of ginsenoside fractions were added to 1/2 MS medium solution and treatment groups with concentrations of 1 mg/L, 10 mg/L, 50 mg/L, and 100 mg/L, respectively, were prepared. Six repeated samples were used for each concentration. The CK group was treated with distilled water. The ginseng hairy roots were inoculated onto different media at 25 °C and cultured for 30 days. The ginseng hairy root samples were then collected and rinsed with deionized water three times. Samples destined for combined phytohormone and transcriptome analysis were promptly flash-frozen in liquid nitrogen and stored at −80 °C. Conversely, samples utilized for physiological index determination and cell viability assays did not require immediate preservation.

#### 4.3.2. Detection of Physiological Indexes of Ginseng Hairy Roots

Biochemical indexes were performed using commercial detection kits for MDA content, SOD activity, and POD activity. The kits were all from Beijing Solarbio Science & Technology Co., Ltd. (Beijing, China).

#### 4.3.3. Detection of Cell Viability of Ginseng Hairy Roots

The root tips of each treatment group were washed with deionized water three times, placed in FDA-PI staining solution [61], and incubated in the dark for 40 min. The samples were then stained in the dark and repeatedly washed with physiological saline for 5 min each time to remove excess dye. The root tips were then sealed with an anti-fluorescence quenching sealing solution and imaged under a laser confocal microscope (STELLARIS 5, Leica, Leica Microsystems Ltd., Heidelberg, Germany.) with excitation and detection wavelengths of 485 nm and 530 nm, respectively.

### 4.4. Phytohormones’ Metabolomic Profiling and Statistical Analysis

The quantification of endogenous phytohormones was performed using an LC-MS/MS platform, which was based on the method described in reference [62]. 

Six parallel samples were pooled as one biological replicate, and three biological replicates were performed. The standard curve for 109 phytohormone-related metabolites was plotted using the concentration and area ratios of the external standard to internal standard as the horizontal and vertical axes. PCA was used to measure sample diversity. Differential fold change was used to screen significantly differential metabolites. The significantly differential endogenous phytohormone metabolic pathways were annotated and enriched using the KEGG database.

### 4.5. RNA-seq Test and Statistical Analysis

RNA-seq analysis was performed as previously described in reference [63]. Briefly, the library was built and checked, followed by the mRNA second-generation sequencing Illumina library and the raw data were filtered using fastp (0.23.2) [64] to obtain clean reads. The parameters were the following: n base limit 15, qualified quality phred 20. Transcript assembly for clean reads was performed using Trinity (v2.13.2) (https://github.com/trinityrnaseq/trinityrnaseq). The parameters were as follows: normalize max read cov 50, min kmer cov 5, and min glue 10. The longest cluster sequence after Corse hierarchical clustering was used as Unigene. The notation information of seven large databases of transcripts was obtained using DIAMOND BLASTX software (v2.0.9). The parameters were as follows: e 1e 5, max hsps 1, and more sensitive k 1. Enrichment analysis was performed based on the hypergeometric test. Gene expression levels were assessed using RSEM software (v1.3.1). Differential gene analysis was performed using DESeq2 (1.22.2). The parameters were FDR < 0.05 and |log2(fold change)|> 1. And functional and biological pathways enriched for differential genes were analyzed using GO and KEGG [63]. To verify the accuracy of the transcriptome data, 12 differentially expressed genes were randomly selected for real-time qPCR analysis.

### 4.6. Integrated Analysis of Metabolomics and Transcriptomics

The differential phytohormone metabolites and differentially expressed genes resulting from the above experiment were jointly analyzed. Pearson’s correlation coefficients of differentially expressed genes and differential phytohormones metabolites were calculated using the COR function of R (4.2.0) and the correlation network was graphed using the R package igraph (1.3.4). The nine quadrants were drawn using the R package ggplot2 (3.3.6). The multiple substances with a Pearson’s correlation coefficient greater than 0.80 and a *p* value less than 0.05 were shown for each differential group by constructing nine quadrants. To mine the correlation of differentially expressed genes and differential phytohormones’ metabolites, the correlation network diagram was drawn. KEGG was used to further explore the relationship between genes and phytohormones. The pathway heat map was plotted for the expression relationships of the most significantly differential phytohormones and representative differential genes.

## 5. Conclusions

This study investigated the molecular mechanism of the polarity and allelopathic autotoxicity of ginsenoside components through an integrated analysis of transcriptomics and targeted endogenous phytohormones metabolomics in plants. The phytohormones cZ, IAM, GA1, and JA-ILE were identified as key response factors to the stress of ginsenoside components with different polarities in ginseng hairy roots. Different-polarity ginsenoside components drove allelopathic autotoxicity by reducing cell viability and regulating the gene expression of key enzymes GID1, KAO, GA20ox, and GA3ox in the GAs’ signaling transduction pathway. The low-polarity ginsenoside component ZG70 had stronger allelopathic autotoxicity and regulated the gene expression of key enzymes in the synthesis pathway of cZ to increase the accumulation of cZ, thereby reducing the overall activity of cytokinins in ginseng hairy roots. In addition, ZG70 regulated the key genes ISS1, TDC, CYP71A13, and CYP83B1, in relation to IAA synthesis pathway, to achieve a differential accumulation of IAM. Meanwhile, ZG70 triggered the DAO gene to activate the IAA oxidative deactivation pathway, thereby cutting auxin signaling transmission and inhibiting the growth of ginseng hairy roots. And, finally, ZG70 can also enhance stress response by regulating the expression of key enzymes in the JA signaling pathway, including JAR1, COI-1, JAZ, and MYC2.

## Figures and Tables

**Table 1:** Transcriptome sequencing of ginseng hairy roots in groups ZG50, ZG70, and CK.

Group	Raw Reads	Clean Reads	Clean Base (G)	Error Rate (%)	Q20 (%)	Q30 (%)	GC Content (%)
CK-1	44,419,584	43,914,206	6.59	0.03	97.49	92.81	43.15
CK-2	48,091,324	46,950,398	7.04	0.03	97.96	94.06	43.22
CK-3	47,199,614	46,069,272	6.91	0.03	97.92	93.95	43.11
ZG50-1	44,735,532	43,812,496	6.57	0.03	97.52	92.92	43.26
ZG50-2	46,572,572	45,450,844	6.82	0.03	97.48	92.83	43.12
ZG50-3	43,992,620	43,192,292	6.48	0.03	97.53	92.93	43.05
ZG70-1	41,996,536	41,172,528	6.18	0.03	97.51	92.87	43.11
ZG70-2	43,724,370	42,959,844	6.44	0.03	97.56	92.95	43.17
ZG70-3	44,232,050	43,265,614	6.49	0.03	97.6	93.08	43.1

**Table 2:** KEGG enrichment analysis data.

Class	KEGG Map	Description	Count Meta	Index Meta	Count Gene
ZG50 vs. CK	ko01100	Metabolic pathways	3	cZ; GA12-ald; GA1	798
ZG50 vs. CK	ko00904	Diterpenoid biosynthesis	3	GA12-ald; GA7; GA1	6
ZG50 vs. CK	ko04075	Phytohormone signal transduction	1	GA1	94
ZG50 vs. CK	ko00908	Zeatin biosynthesis	1	cZ	5
ZG50 vs. CK	ko01110	Biosynthesis of secondary metabolites	4	cZ; GA12-ald; GA7; GA1	390
ZG70 vs. CK	ko01110	Biosynthesis of secondary metabolites	3	cZ; GA12-ald; GA7	691
ZG70 vs. CK	ko01100	Metabolic pathways	3	IAM; cZ; GA12-ald	1156
ZG70 vs. CK	ko00904	Diterpenoid biosynthesis	2	GA12-ald; GA7	15
ZG70 vs. CK	ko00908	Zeatin biosynthesis	1	cZ	14
ZG70 vs. CK	ko00380	Tryptophan metabolism	1	IAM	28
ZG70 vs. ZG50	ko04075	Phytohormone signal transduction	2	GA1; JA-ILE	305
ZG70 vs. ZG50	ko01110	Biosynthesis of secondary metabolites	2	cZ; GA1	1136
ZG70 vs. ZG50	ko01100	Metabolic pathways	2	cZ; GA1	2070
ZG70 vs. ZG50	ko00904	Diterpenoid biosynthesis	1	GA1	22
ZG70 vs. ZG50	ko00908	Zeatin biosynthesis	1	cZ	19

## Data Availability

Data are contained within the article and Supplementary Materials.

## Supplementary Materials

The following supporting information can be downloaded at https://www.mdpi.com/article/10.3390/molecules29081877/s1. Table S1: The composition and contents of ginsenosides in ginseng fractions with different polarities; Table S2: The 109 endogenous phytohormones and their metabolites detected by LC-MS/MS; Table S3: The content of 52 endogenous phytohormones and their metabolites detected by LC-MS/MS; Table S4: The qPCR validation of 12 key genes’ data; Figure S1: Basic peak ion current chromatogram of different-polarity ginsenoside components; Figure S2: The extracted ion current chromatogram of 109 endogenous phytohormones. (A) Negative ion mode. (B) Positive ion mode; Figure S3: Chord chart of KEGG enrichment of ZG50 vs. CK group; Figure S4: Chord chart of KEGG enrichment of ZG70 vs. CK group; Figure S5: Circle plot of GO enrichment of ZG50 vs. CK group; Figure S6: Circle plot of GO enrichment of ZG70 vs. CK group.
