# Supplementary material for: Analysis Transcriptome and Phytohormone Changes Associated with the Allelopathic Effects of Ginseng Hairy Roots Induced by Different-Polarity Ginsenoside Components
Source: Molecules. 2024 Apr 19;29(8):1877. doi: 10.3390/molecules29081877 (PMC11053915; doi:10.3390/molecules29081877)
Supplement: Supplementary file 1 [file molecules-29-01877-s001.zip › molecules-2955247-supplementary.pdf]

Table S1 The composition and contents of ginsenosides in ginseng fractions with different polarity.

Ginsenosides	Type	t_R	$[M\text{-}H]^-$ (*m/z*)	Standard Curve	Content (mg/g)		
					ZG50	ZG70	ZG100
NotoR_1	PPT	5.29	931.51	y=1064.9x+44782, R^2=0.9996	0.07	0	0
Re	PPT	6.24	945.58	y=1119.4x+212959, R^2=0.9999	0.81	0.18	0.08
Rg_1	PPT	6.58	799.30	y=175.04x+129853, R^2=0.9991	0.68	0.11	0.05
Rf	PPT	12.83	799.51	y=3243.4x+1492004.5, R^2=0.9990	0	0.58	0.03
Rb_1	PPD	14.50	1107.73	y=1222915.4x+914658.6, R^2=0.9990	0.06	1.39	0.11
NotoR_2	PPT	14.90	769.48	y=696.98x–67079, R^2=0.9998	0	0.64	0.03
Ro	OLE	15.30	955.32	y=2425.1x-14893, R^2=0.9994	0.52	0.09	0.06
Rg_2	PPT	15.36	783.54	y=5249.1x-7583.1, R^2=0.9986	0	0.13	0
Rc	PPD	15.74	1077.68	y=1311.1x+1681931.9, R^2=0.9953	0.01	0.92	0.06
Rb_3	PPD	15.89	1077.34	y=1045244.7x+18750.1, R^2=0.9996	0	0.39	0.02
Rh_1	PPT	16.45	683.49	y=3719.8x-695.04, R^2=0.9992	0	0.0004	0.0002
Rb_2	PPD	17.03	1077.75	y=1349.6x+979755, R^2=0.9992	0.01	1.29	0.07
Rd	PPD	19.38	945.60	y=1644956.7x+1417481.6, R^2=0.9988	0	0.63	0.05
Rg_3	PPD	20.90	783.31	y=10852.9x-207220.7, R^2=0.9960	0.0004	0.0007	0.0009
F_2	PPD	20.93	783.34	y=26842x–20104, R^2=0.9905	0.00002	0.00006	0.0003

Table S2 The 109 endogenous phytohormones and their metabolites detected by LC-MS/MS.

No.	Phytohormones	Class	LLOQ	ULOQ	RT	Equation	r
1	Abscisic acid (ABA)	ABA	0.1	500	5.31	$y=0.1011x+2.4879e^{-4}$	0.9987
2	ABA-glucosyl ester (ABA-GE)	ABA	5	500	4.49	y=0.4310x+0.0069	0.9996
3	Abscisic aldehyde (ABA-ald)	ABA	5	500	5.54	$y=0.0199x-9.7725e^{-5}$	0.9986
4	Indole-3-carboxylic Acid (ICA)	Auxin	0.5	500	4.78	$y=0.0099x+5.6203e^{-4}$	0.9997
5	Indole-3-carboxaldehyde (ICAld)	Auxin	0.1	500	4.93	y=0.0438x+0.0381	0.9959
6	3-Indolebutyric acid (IBA)	Auxin	0.2	500	5.79	y=0.0234x+0.1355	0.9999
7	Indole-3-acetic acid (IAA)	Auxin	0.2	500	5.05	$y=0.0142x+5.8495e^{-4}$	0.9999
8	1-O-indol-3-ylacetylglucose (IAA-Glc)	Auxin	5	10000	4.41	y=0.1570x-0.0036	0.9964
9	Indoleacetyl glutamic acid (IAA-Glu)	Auxin	0.1	500	4.41	y=0.1570x-0.0036	0.9964
10	3-Indoleacetonitrile (IAN)	Auxin	0.5	500	4.30	y=0.0041x+0.0017	0.9955
11	Indole-3-acetyl glycine (IAA-Gly)	Auxin	1	500	4.44	y=0.2916x+0.0191	0.9999
12	2-oxindole-3-acetic acid (OxIAA)	Auxin	1	500	4.24	$y=0.0112x+5.4380e^{-4}$	0.9995
13	Indole-3-acetyl-L-aspartic acid (IAA-Asp)	Auxin	0.1	500	4.30	$y=0.0183x+2.1655e^{-4}$	0.9992
14	N-(3-Indolylacetyl)-L-leucine (IAA-Leu)	Auxin	0.1	500	5.79	y=0.9413x+0.0290	0.9997
15	N-(3-Indolylacetyl)-L-valine (IAA-Val)	Auxin	0.1	500	5.42	y=0.7679x+0.0011	0.9998
16	Indole-3-acetyl-L-tryptophan (IAA-Trp)	Auxin	0.1	500	5.75	y=0.3439x+0.0034	0.9999
17	N-(3-Indolylacetyl)-L-alanine (IAA-Ala)	Auxin	0.1	500	4.74	y=0.2700x+0.0063	0.9997
18	N-(3-Indolylacetyl)-L-phenylalanine (IAA-Phe)	Auxin	0.5	500	5.87	y=0.5974x+0.0082	0.9981
19	Indole-3-acetyl-Lglutamic acid dimethyl ester (IAA-Glu-diMe)	Auxin	0.5	500	5.59	$y=0.0416x-5.3100e^{-4}$	0.9983
20	Indole-3-acetyl-L-leucine methyl ester (IAA-Leu-Me)	Auxin	0.1	500	6.48	y=2.0010x+0.0208	0.9957
21	Indole-3-acetyl-L-valine methyl ester (IAA-Val-Me)	Auxin	0.1	500	6.15	y=1.9089x+0.0185	0.9998
22	Indole-3-acetyl-L-phenylalanne methyle ester (IAA-Phe-Me)	Auxin	0.1	500	6.52	y=1.2831x+0.0091	0.9995
23	3-Indoleacetamide (IAM)	Auxin	0.1	500	4.34	y=0.4887x+0.0084	0.9966
24	Tryptamine (TRA)	Auxin	0.1	500	3.63	y=0.1381x+0.0068	0.9988
25	Indole-3-lactic acid (ILA)	Auxin	2	500	4.65	y=0.0170x+0.0017	0.9990
26	3-Indoleacrylic acid (IA)	Auxin	0.2	500	5.27	$y=0.0136x+5.9659e^{-4}$	0.9990
27	L-tryptophan (TRP)	Auxin	2	10000	3.35	y=0.0066x+0.0060	0.9992
28	Methyl indole-3-acetate (MEIAA)	Auxin	0.2	500	6.12	y=0.0775x+0.0013	0.9915
29	3-Indolepropionic acid (IPA)	Auxin	0.1	500	5.48	y=0.0395x+0.0010	0.9995
30	Indole	Auxin	0.5	500	N/A	y=0.0048x+0.0032	0.9992
31	N6-isopentenyladenine (IP)	CK	0.1	500	4.14	y=0.0701x+0.0078	0.9967
32	trans-Zeatin (tZ)	CK	0.1	500	3.06	y=0.1707x+0.0031	0.9996
33	cis-Zeatin (cZ)	CK	0.1	500	3.26	y=0.0752x+0.0017	0.9998
34	Dihydrozeatin (DZ)	CK	0.1	500	3.18	y=0.0756x+0.0014	0.9993

35	Dihydrozeatin ribonucleoside (DHZR)	CK	0.1	500	3.63	y=0.0819x+0.0013	0.9904
36	cis-Zeatin riboside (cZR)	CK	0.1	500	3.70	$y=0.1016x+7.5735e^{-4}$	0.9905
37	cis-Zeatin-9-glucoside (cZ9G)	CK	0.1	500	3.32	y=0.4104x+0.0025	0.9904
38	N6-Isopentenyl-adenine-9-glucoside (iP9G)	CK	0.1	500	4.11	y=0.2335x+0.0037	0.9927
39	N6-Isopentenyl-adenine-7-glucoside (iP7G)	CK	0.1	500	3.72	y=0.4002x+0.0040	0.9921
40	trans-Zeatin-O-glucoside (tZOG)	CK	0.2	500	2.92	$y=0.1019x-1.3589e^{-4}$	0.9995
41	Dihydrozeatin-O-glucoside riboside (DHZROG)	CK	0.1	500	3.52	y=0.3861x+0.0035	0.9998
42	cis-Zeatin-O-glucoside riboside (cZROG)	CK	0.1	500	3.54	$y=0.0237x+1.7623e^{-4}$	0.9967
43	4-[[(9-beta-D-Glucopyranosyl-9H-purin-6-yl)amino]methyl]phenol (pT9G)	CK	0.1	500	3.59	y=0.1443x+0.0012	0.9931
44	2-Chloro-trans-zeatin (2CltZ)	CK	0.1	500	4.45	$y=0.1209x+2.1126e^{-4}$	0.9989
45	para-Topolin (pT)	CK	0.2	500	3.57	y=0.0622x+0.0016	0.9980
46	meta-Topolin (mT)	CK	2	500	3.74	y=0.1035x+0.0012	0.9909
47	meta-Topolin riboside (mTR)	CK	0.1	500	4.11	y=0.2736x+0.0029	0.9900
48	ortho-Topolin (oT)	CK	0.1	500	4.16	y=0.1806x+0.0020	0.9901
49	6-Benzyladenine (BAP)	CK	0.1	500	4.33	y=0.1751x+0.0040	0.9919
50	6-Benzyladenosine (BAPR)	CK	0.1	500	4.68	y=0.4545x+0.0039	0.9915
51	Kinetin (K)	CK	0.1	500	3.82	y=0.0815x+0.0014	0.9953
52	Kinetin riboside (KR)	CK	0.1	500	4.25	y=0.2748x+0.0042	0.9906
53	para-Topolin riboside (pTR)	CK	0.1	500	3.97	y=0.1858x+0.0031	0.9907
54	ortho-Topolin riboside (oTR)	CK	0.1	500	4.49	y=0.1799x+0.0017	0.9911
55	meta-Topolin-9-glucoside (mT9G)	CK	0.1	500	3.75	y=0.1487x+0.0022	0.9907
56	ortho-Topolin-9-glucoside (oT9G)	CK	0.1	500	4.12	$y=0.1170x+5.5352e^{-4}$	0.9910
57	N6-Benzyladenine -9-glucoside (BAP9G)	CK	0.1	500	4.28	y=0.3662x+0.0033	0.9906
58	N6-Benzyladenine-7-glucoside (BAP7G)	CK	0.1	500	3.82	y=0.4782x+0.0071	0.9904
59	Kinetin-9-glucoside (K9G)	CK	0.1	500	3.83	y=0.2378x+0.0037	0.9920
60	2-Methylthio-N6-isopentenyladenine (2MeSiP)	CK	0.1	500	5.90	$y=0.0226x+5.4621e^{-4}$	0.9900
61	2-methylthio-cis-zeatin (2MeScZ)	CK	0.1	500	4.54	$y=0.0204x+4.02684e^{-4}$	0.9922
62	2-Methylthio-cis-zeatin riboside (2MeScZR)	CK	0.1	500	4.60	y=0.1306x+0.0033	0.9921
63	2-Methylthio-N6-isopentenyladenosine (2MeSiPR)	CK	0.1	500	5.79	$y=0.0269x-3.8687e^{-5}$	0.9908
64	N6-isopentenyladenosine (IPR)	CK	0.1	500	4.54	y=0.0860x+0.0012	0.9906
65	trans-Zeatin riboside (tZR)	CK	0.1	500	3.61	y=0.1534x+0.0019	0.9957
66	Dihydrozeatin-7-glucoside (DHZ7G)	CK	0.1	500	3.20	y=0.3791x+0.0018	0.9934
67	cis-Zeatin riboside monophosphate (cZRMP)	CK	2	500	3.02	y=0.0457x-0.0011	0.9983
68	N-6-iso-pentenyladenosine-5'-monophosphate (iPRMP)	CK	1	500	3.83	$y=0.1100x-6.2032e^{-4}$	0.9974
69	trans-Zeatin-9-glucoside (tZ9G)	CK	1	500	3.19	y=0.0758x+0.0045	0.9990
70	9-Ribosyl-trans-zeatin 5'-monophosphate (tZRMP)	CK	2	500	2.41	$y=0.0293x-5.2890e^{-4}$	0.9950

71	1-Aminocyclopropanecarboxylic acid (ACC)	ETH	1	500	0.72	$y=1.7969e^4x+2.6108e^4$	0.9959
72	Gibberellin A1 (GA1)	GA	2	500	4.49	y=0.0216x+0.0147	0.9937
73	Gibberellin A3 (GA3)	GA	1	500	4.46	y=0.0634x+0.0049	0.9974
74	Gibberellin A4 (GA4)	GA	2	500	6.19	y=0.0270x+0.0070	0.9914
75	Gibberellin A7 (GA7)	GA	1	500	6.11	y=0.2677x+0.0234	0.9977
76	Gibberellin A9 (GA9)	GA	2	500	6.91	y=0.0679x+0.0056	0.9913
77	Gibberellin A15 (GA15)	GA	2	500	6.91	y=0.0221x+0.0011	0.9948
78	Gibberellin A19 (GA19)	GA	5	500	5.17	y=0.0294x+0.0092	0.9955
79	Gibberellin A20 (GA20)	GA	2	500	5.37	y=0.0176x+0.0025	0.9940
80	Gibberellin A24 (GA24)	GA	2	500	6.37	y=0.0526x+0.0015	0.9941
81	Gibberellin A53 (GA53)	GA	2	500	5.76	y=0.0400x+0.0153	0.9997
82	Gibberellin A44 (GA44)	GA	5	500	5.41	$y=0.0216x+9.0282e^{-4}$	0.9936
83	Gibberellin A8 (GA8)	GA	2	500	3.83	y=0.0978x+0.0035	0.9953
84	Gibberellin A5 (GA5)	GA	5	500	5.30	y=0.0330x+0.0041	0.9987
85	Gibberellin A6 (GA6)	GA	1	500	4.90	y=0.0797x+0.0039	0.9962
86	Gibberellin A29 (GA29)	GA	2	500	3.99	y=0.0042x+0.0031	0.9964
87	Gibberellin A34 (GA34)	GA	2	500	5.57	y=0.0838x+0.0025	0.9980
88	Gibberellin A51 (GA51)	GA	5	500	5.71	y=0.0138x+0.0053	0.9901
89	Gibberellin A12 aldehyde (GA12-ald)	GA	5	500	7.76	y=0.0230x+0.0015	0.9958
90	Jasmonic Acid (JA)	JA	0.2	500	5.83	y=0.0267x+0.0014	0.9967
91	Methyl jasmonate (MEJA)	JA	0.2	500	6.96	y=0.4514x+0.0137	0.9999
92	Dihydrojasmonic acid (H2JA)	JA	1	10000	6.20	y=0.0360x+0.0059	0.9993
93	Jasmonoyl-L-isoleucine (JA-ILE)	JA	0.1	500	6.37	y=0.0600x+0.0012	0.9922
94	cis(+)-12-Oxophytodienoic acid (OPDA)	JA	0.1	500	7.30	y=0.3276x+0.0122	0.9984
95	N-[(-)-Jasmonoyl]-(l)-phenalanine (JA-Phe)	JA	0.1	500	6.41	y=0.1445x+0.0029	0.9984
96	N-[(-)-Jasmonoyl]-(L)-valine (JA-Val)	JA	0.1	500	6.03	y=0.2704x+0.0040	0.9902
97	3-oxo-2-(2-(Z)-Pentenyl)cyclopentane-1-butyric acid (OPC-4)	JA	2	500	6.44	y=0.0143x+0.0169	0.9931
98	3-oxo-2-(2-(Z)-Pentenyl)cyclopentane-1-hexanoic acid (OPC-6)	JA	5	500	7.06	y=0.0225x+0.0161	0.9966
99	12-Hydroxyjasmonic acid (12-OH-JA)	JA	5	500	4.81	y=0.0342x+0.0442	0.9976
100	Jasmonate-1-aminocyclopropane-1-carboxylic acid (JA-ACC)	JA	0.5	500	5.37	y=0.0459x+0.0017	0.9904
101	Melatonine (MLT)	MLT	0.5	500	4.90	$y=9.0017e^5x+1.3671e^4$	0.9931
102	Salicylic Acid (SA)	SA	0.5	500	5.10	y=0.1037x+0.0207	0.9997
103	Salicylic acid 2-O-β-Glucoside (SAG)	SA	1	10000	3.59	y=0.0309x+0.0092	0.9926
104	L-Phenylalanine (Phe)	SA	1	15000	2.00	$y=718.2031x+2.0048e^4$	0.9919
105	trans-Cinnamic acid (t-CA)	SA	200	10000	5.59	y=235.6716x+920.8265	0.9990
106	2-Coumarate	SA	10	500	4.90	$y=6.3798e^4x+1.6791e^4$	0.9986

107	2-Methoxycarbonylphenyl beta-D-glucopyranoside (MeSAG)	SA	20	500	4.17	$y=3.8563e^4x+2.3844\ e^4$	0.9995
108	5-Deoxystrigol (5DS)	SL	2	10000	7.54	$y=6.2364e^3x+832.0683$	0.9990
109	(±)Strigol (ST)	SL	20	10000	6.42	y=579.6571x+207.0285	0.9970

Table S3 The content of 52 endogenous phytohormones and their metabolites detected by LC-MS/MS.

Index	N	M ± SE (ng/g)		
		**CK**	**ZG70**	**ZG50**
12-OH-JA	3	32.578±1.265	32.840±2.148	48.749±3.087
2MeScZR	3	0.137±0.009	0.348±0.0129	0.136±0.009
ABA	3	1.521± 0.069	1.389±0.079	1.284±0.105
ABA-ald	3	41.911±0.756	56.436±3.843	45.895±4.832
ABA-GE	3	0.491±0.023	0.384±0.042	0.472±0.049
ACC	3	48.349±1.554	45.939±1.744	40.287±3.799
BAPR	3	0.272±0.024	0.255±0.021	0.219±0.026
cZ	3	0.019±0.005	0.170±0.004	0
cZ9G	3	0.072±0.007	0	0
cZR	3	3.167±0.064	4.043±0.156	2.228±0.011
cZRMP	3	1.681±0.035	4.168±0.085	1.604±0.105
DHZR	3	0.110±0.023	0.116±0.012	0.096±0.024
DHZROG	3	0.131±0.021	0.204±0.028	0.146±0.009
GA1	3	3.432±0.776	3.881±1.012	0
GA12-ald	3	0	0.499±0.024	0.344±0.020
GA15	3	3.349±0.236	5.404±0.099	3.134±0.270
GA19	3	4.254±0.409	3.513±0.151	4.230±0.303
GA29	3	53.917±5.104	52.062±4.892	54.352±1.483
GA53	3	0.628±0.068	0.875±0.149	0.464±0.126
GA7	3	0	0.020±0.008	0.014±0.001
GA8	3	4.863±0.168	5.037±0.168	5.161±0.293
IAA	3	29.208±0.236	42.622±1.245	28.703±1.456
IAA-Asp	3	237.820±9.239	280.040±5.529	227.340±23.144
IAA-Glc	3	3.322±0.058	3.380±0.773	5.978±0.748
IAA-Glu	3	1.764±0.140	2.185±0.170	2.343±0.522
IAA-Phe	3	0.285±0.018	0.475±0.039	0.282±0.030
IAA-Trp	3	0.529±0.054	0.701±0.036	0.534±0.043
IAM	3	0.253±0.035	0.747±0.082	0.375±0.076
IAN	3	0.644±0.088	0.617±0.078	0.471±0.046
ICA	3	1.606±0.180	3.938±0.118	2.416±0.271
ICAld	3	5.611±0.422	6.851±0.809	6.805±0.354
ILA	3	0.984±0.016	0.769±0.073	0
Indole	3	1689.300±49.286	1502.400±116.150	1763.100±166.810
IP	3	0.129±0.008	0.180±0.011	0.154±0.013

IPR	3	0.233±0.010	0.248±0.014	0.243±0.026
iPRMP	3	0.669±0.018	1.097±0.073	0.933±0.034
JA	3	8.983±0.428	9.986±0.153	8.742±0.709
JA-ILE	3	7.395±0.322	13.615±0.117	6.367±0.310
JA-Val	3	0.136±0.002	0.262±0.012	0.120±0.012
KR	3	0.330±0.037	0.467±0.033	0.536±0.027
MEIAA	3	2.713±0.093	2.536±0.060	2.401±0.054
OPC-6	3	44.584±1.748	139.500±14.321	106.720±9.946
OPDA	3	2.165±0.142	0	2.998±0.024
oT9G	3	0.102±0.020	0	0
OxIAA	3	217.360±2.195	315.220±17.316	270.34±20.702
Phe	3	2998.100±73.567	3656.200±109.220	2889.100±66.487
pT9G	3	0	0.661±0.053	0.988±0.063
SA	3	25.125±1.622	38.755±0.759	27.774±2.776
SAG	3	1281.400±78.424	3476.300±107.350	2527.600±26.568
TRA	3	0.251±0.025	0.229±0.043	0.227±0.012
TRP	3	12777.000±154.380	11312.000±327.710	10825.000±120.720
tZRMP	3	1.406±0.071	1.730±0.072	1.233±0.137

Table S4 The qPCR validation of 12 key genes data.

Gene	Group	Mean Expression (2-ΔΔCt)	STDEV	Primer sequence (5' to 3')	Product size (bp)
TRIT1	CK	1.000	0.005	TGTTGCACTACATGATTGATGATCC	91
	ZG70	0.399	0.032	TGTGCATAACAAACAAAGTCTGAAC	
cisZOG	CK	1.003	0.091	ATTTCATCGCCCACATCCGT	184
	ZG70	0.647	0.074	ACGATACCAAGACAACGCCA	
CPS	CK	1.004	0.112	GCTGTTGTCCCATCCTCACT	174
	ZG70	0.697	0.036	GAGTCGCATAGCACCAGTTG	
CYP71A13	CK	1.004	0.107	CAAGACCTCCTGAGCTGCC	118
	ZG70	0.185	0.029	ATTACCGCATCGCTCACTCC	
CYP83B1	CK	1.001	0.058	GGGATTAGCATGGGAGTTGCT	127
	ZG70	0.397	0.010	AGGTTAGACCAGGCAGTGAG	
DAO	CK	1.001	0.060	ATCTACGCCCAAGCTCTCCA	195
	ZG70	0.366	0.026	TATCCAGCGGTGCCACTCTA	
GST	CK	1.004	0.107	AGCCAAATTCATGCGGTCTG	101
	ZG70	1.532	0.178	GGAAGCCCGGAACAAAGAAAC	
GID1	CK	1.002	0.077	TCTCTTTCTGTCATGCTCTCCA	190
	ZG50	0.458	0.021	TTCCAGTACCTTGTATGCCTGT	
GA20ox	CK	1.001	0.058	GAGAGGAAGCCGCCTAAGTC	163
	ZG50	2.596	0.186	TGTCTCCAGCACCCAAAGAC	
GA3ox	CK	1.009	0.162	CCTCAGCACTGCCGGAATTA	121
	ZG50	12.146	1.079	AAAGTTCATCCCGTCCACCA	
HSP20	CK	1.000	0.025	CCGGTGAACTGACAGAGTTG	159
	ZG50	0.493	0.009	CACACGAACGATATTCAAGCG	
groEL	CK	1.000	0.028	CGTGGTACGCTTTGTCTCCT	133
	ZG50	0.173	0.015	TGTCTTCGGTTGGTTCCCTG	

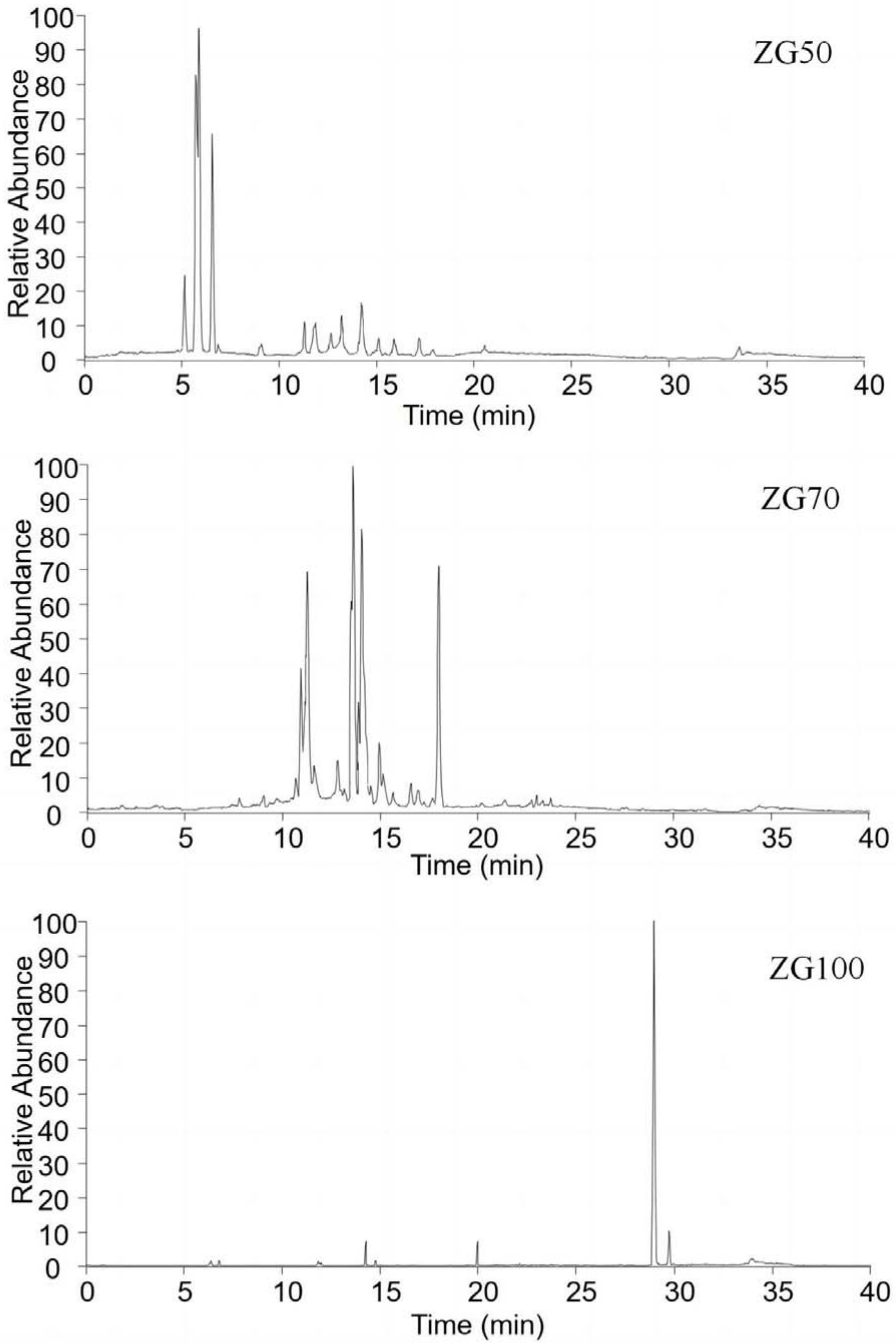

Figure S1 Basic peak ion current chromatogram of different polarity ginsenoside components.

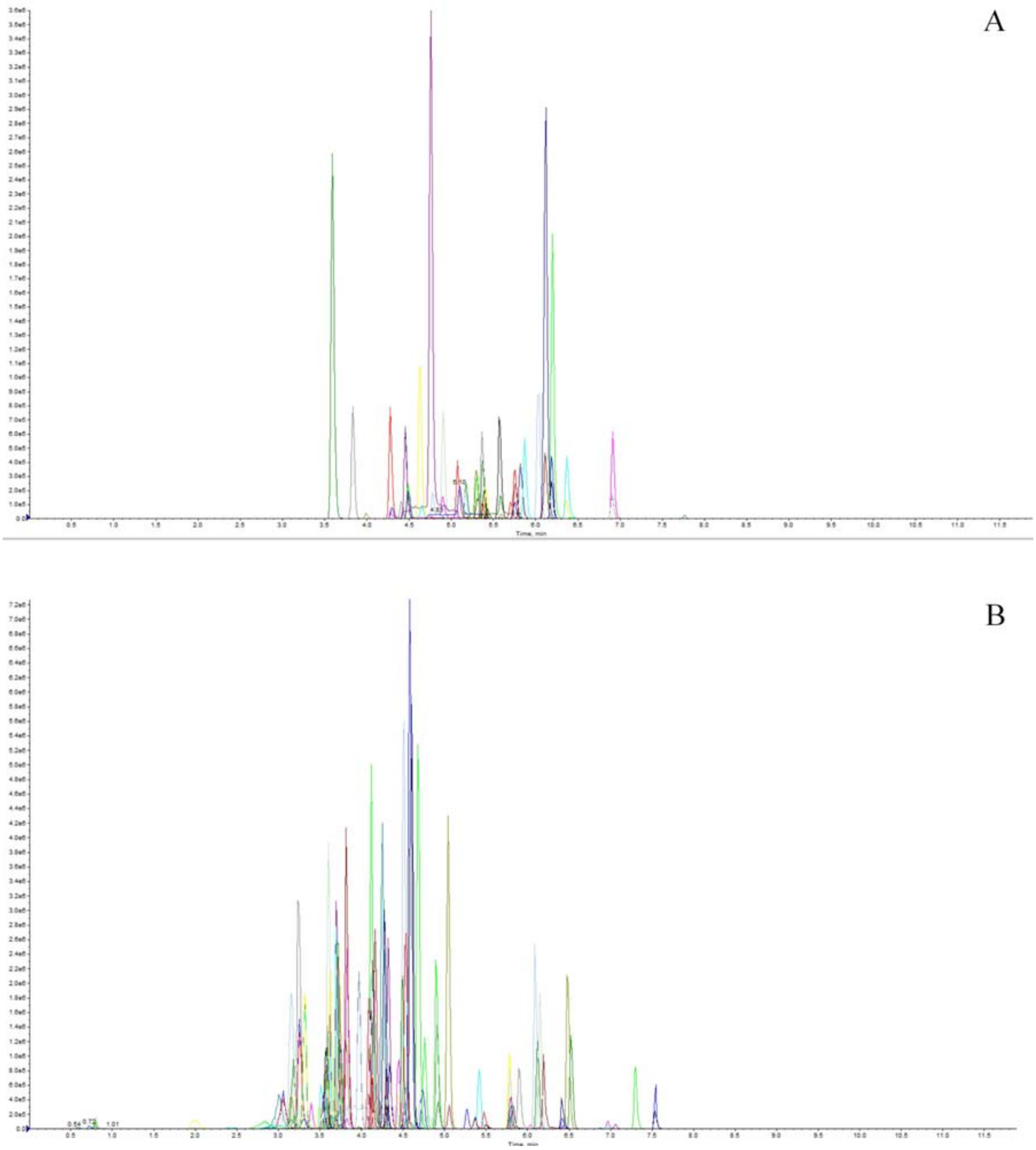

Figure S2 The extracted ion current chromatogram of 109 endogenous phytohormones. (A) Negative ion mode. (B) Positive ion mode.

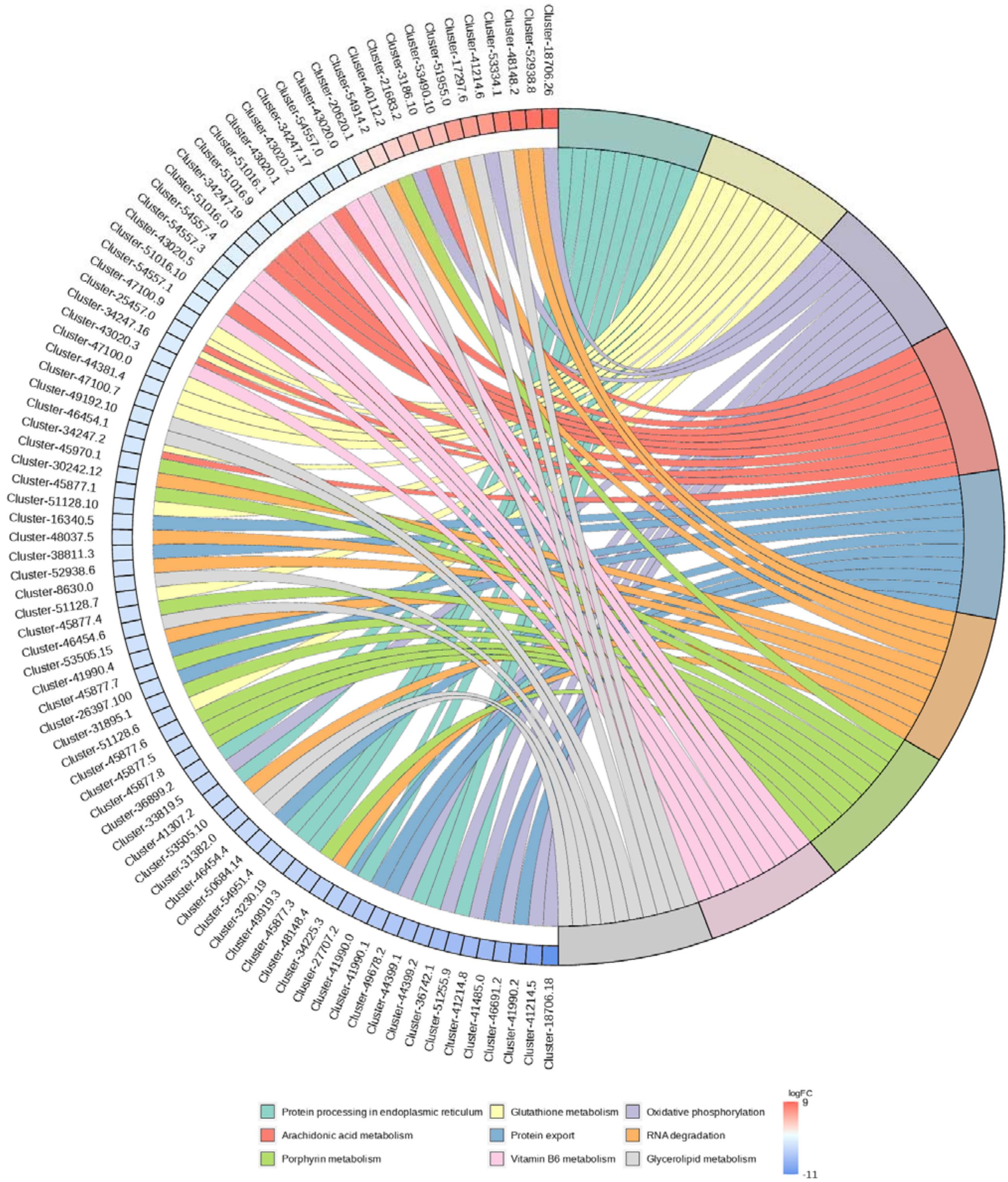

Figure S3 Chord chart of KEGG enrichment of ZG50 VS CK group.

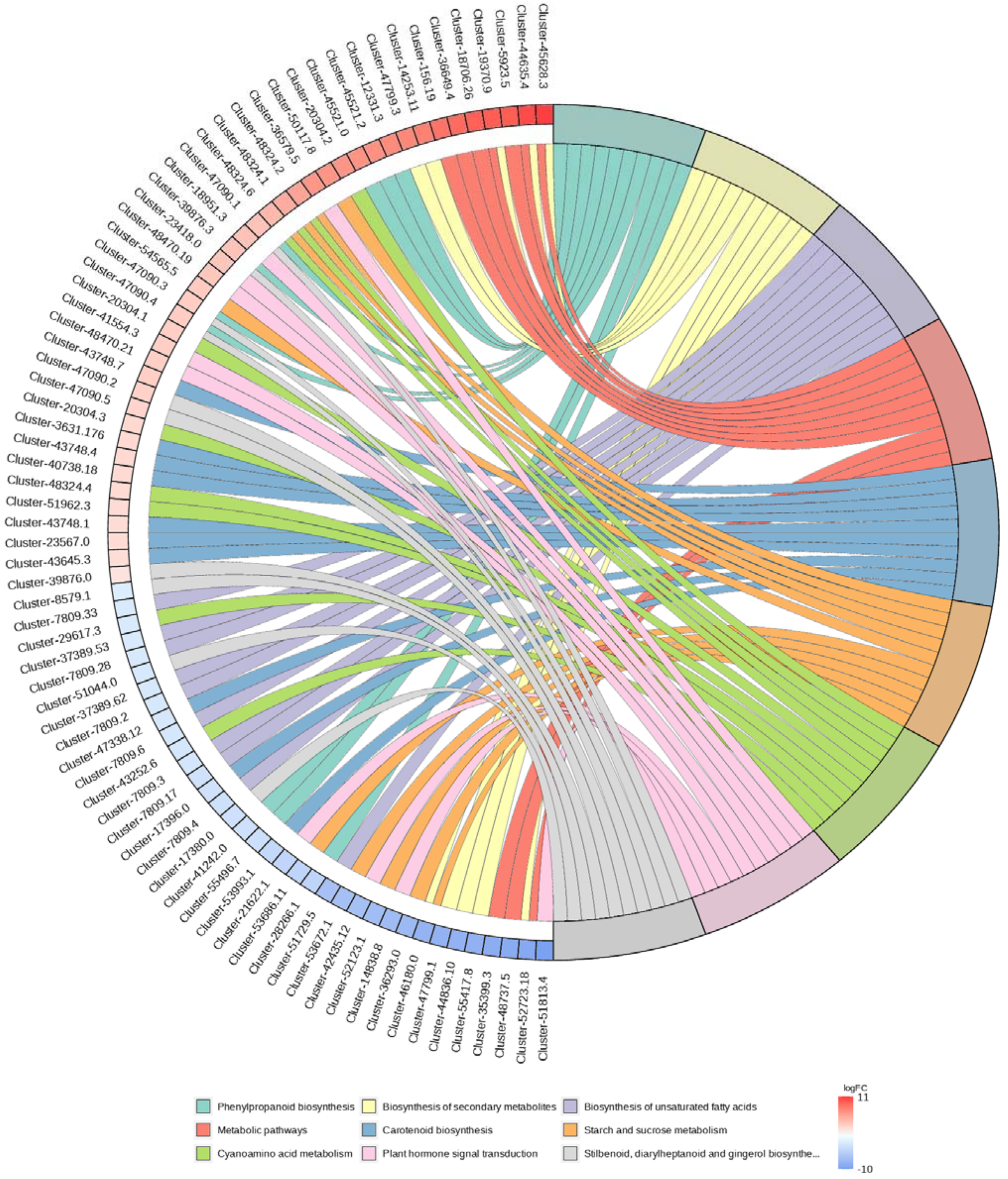

Figure S4 Chord chart of KEGG enrichment of ZG70 VS CK group.

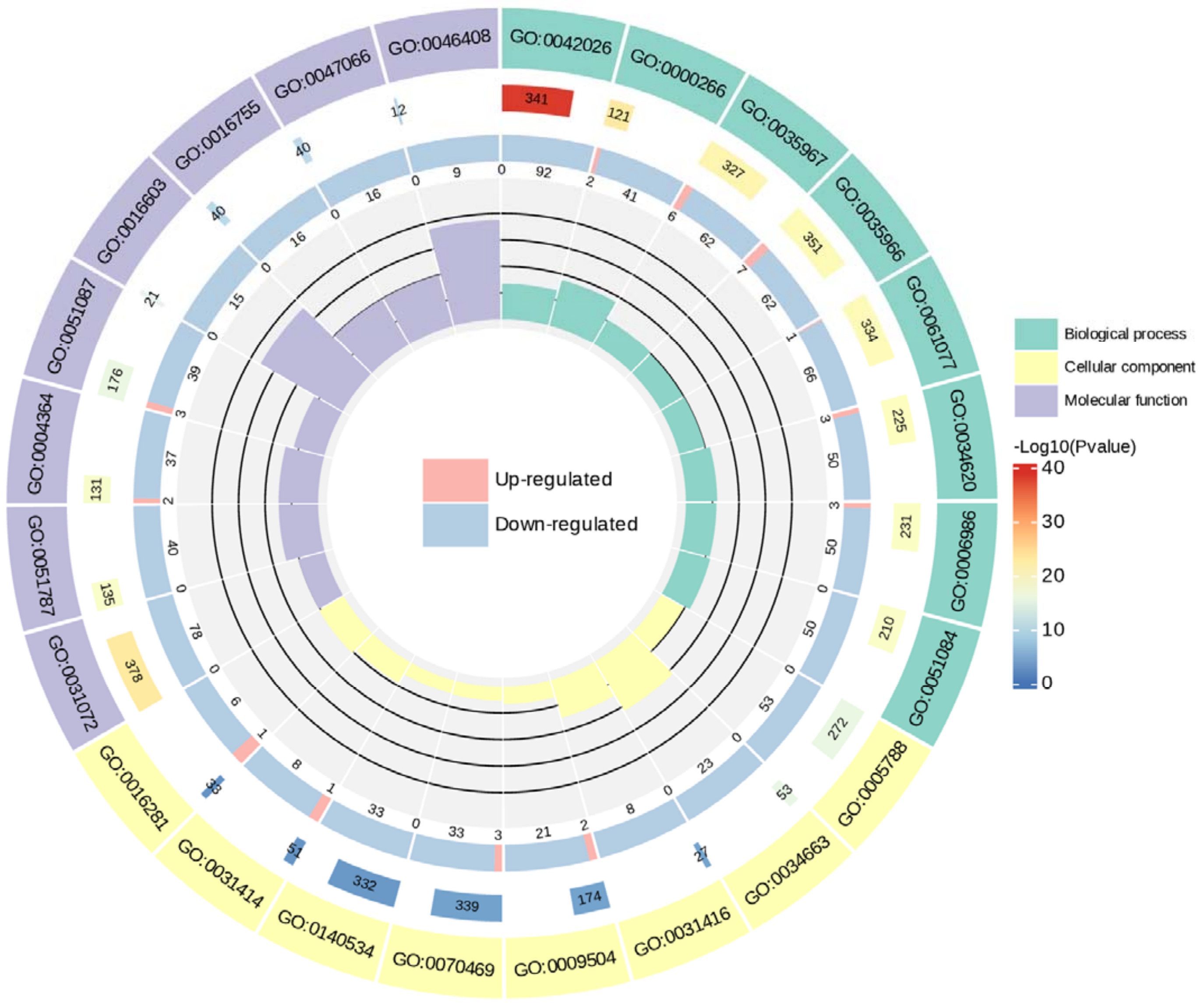

Figure S5 Circle plot of GO enrichment of ZG50 VS CK group.

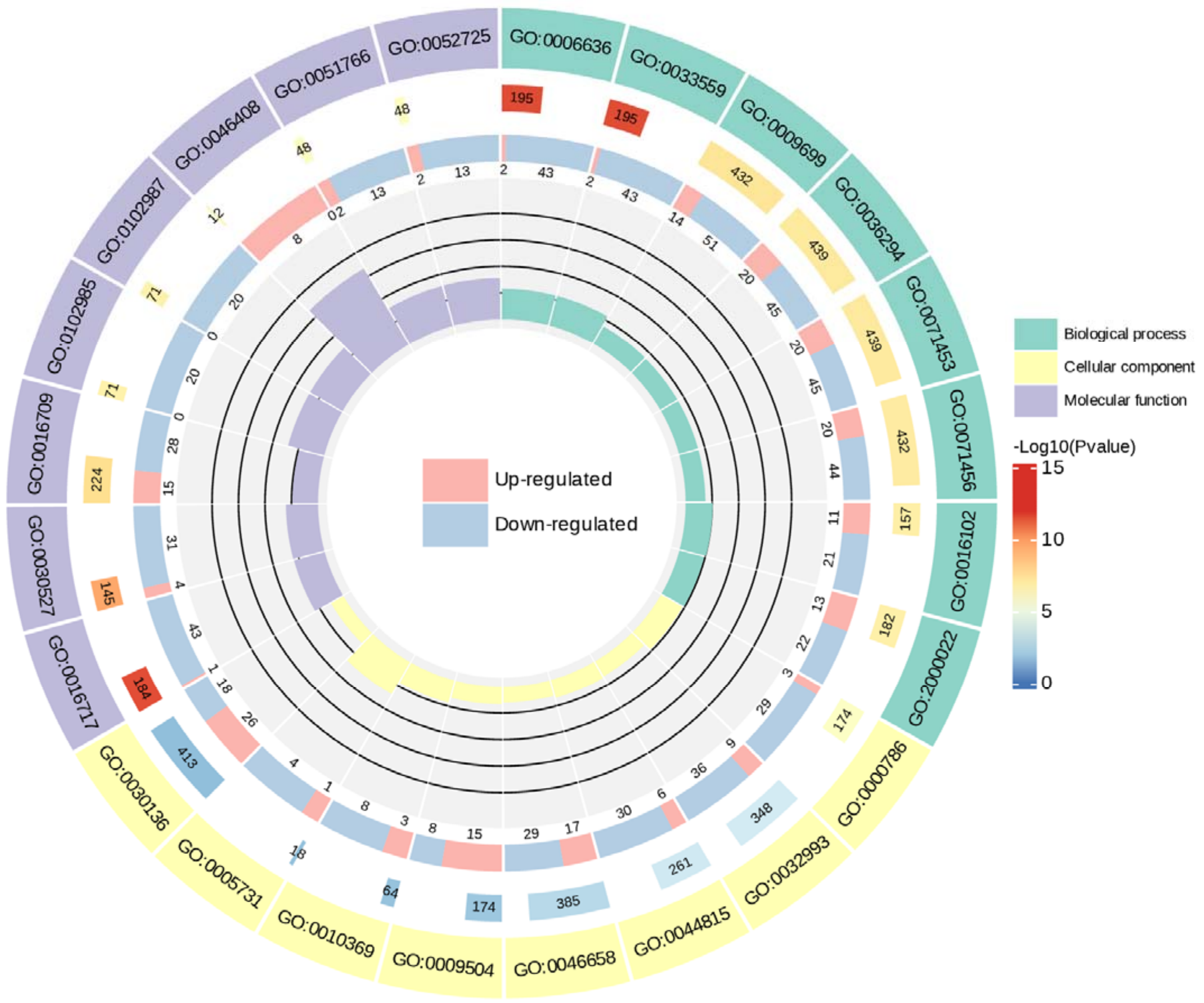

Figure S6 Circle plot of GO enrichment of ZG70 VS CK group.
